# The Impact of COVID-19 Pandemic on Dermatological Conditions: A Novel, Comprehensive Review

**DOI:** 10.3390/dermatopathology9030027

**Published:** 2022-06-29

**Authors:** Gehan A. Pendlebury, Peter Oro, William Haynes, Drew Merideth, Samantha Bartling, Michelle A. Bongiorno

**Affiliations:** 1 College of Osteopathic Medicine, Nova Southeastern University, Fort Lauderdale, FL 33314, USA; 2 School of Osteopathic Medicine in Arizona, A.T. Still University, Mesa, AZ 85206, USA; peter.oro@atsu.edu (P.O.); bill.haynes@atsu.edu (W.H.); sa202621@atsu.edu (D.M.); 3 Department of Dermatology, Walter Reed National Military Medical Center, Bethesda, MD 20814, USA; samantha.j.bartling.mil@mail.mil (S.B.); michelle.a.bongiorno.mil@mail.mil (M.A.B.)

**Keywords:** COVID-19, COVID-19 pandemic, SARS-CoV-2 infection, cutaneous manifestations, COVID arm, pandemic psychosocial stress, personal protective equipment, COVID vaccinations, psychodermatology, teledermatology

## Abstract

*Background:* The earliest cases of Severe Acute Respiratory Syndrome Coronavirus 2 (SARS-CoV-2) emerged in Wuhan, China, in December 2019. Since the declaration as a pandemic on 11 March 2020, further dermatological conditions continue to be documented. We herein present a novel literature review of dermatological manifestations associated with the Coronavirus Disease 2019 (COVID-19) pandemic. To date, this literature review is the first broad-spectrum examination that analyzes a range of dermatological manifestations related to the COVID-19 pandemic: infection, vaccinations, personal protective equipment (PPE), and psychosocial factors. *Methods:* A detailed literature search was conducted using key terms for cutaneous manifestations associated with the scope of this review. The search retrieved 2199 articles. *Results:* The COVID-19 pandemic has triggered a significant range of dermatologic sequela. Etiologies of lesions continue to be investigated. Proposed mechanisms include inflammatory response to spike protein, vitamin D deficiency, ACE2 receptor activation, androgen levels, and increased psychological stress. One prominent mechanism describes viral spike protein invasion into the dermis by binding to the angiotensin-converting enzyme 2 (ACE-2) receptors in keratinocytes, with a secondary immunological response. *Conclusions:* Dermatologists play an integral role in the proper diagnosis and treatment of COVID-related lesions. Early treatment regimens and timely prophylaxis have been shown to safely reduce infection-related dermatological sequelae. Additional investigations and data collection can reduce disease burden and improve overall prognosis.

## 1. Introduction

Infection caused by SARS-CoV-2 has a high transmission rate with various associated sequelae. The first documented cases of Coronavirus disease 2019 (COVID-19) appeared in Wuhan, China, in December 2019 [1]. The World Health Organization (WHO) declared the COVID-19 outbreak a global pandemic on 11 March 2020 [2]. Severe Acute Respiratory Syndrome Coronavirus 2 (SARS-CoV-2) has become an international health concern. As of 1 June 2022, 540,470,607 cases of SARS-CoV-2 infections have been reported worldwide, with a total of 6,333,332 deaths and 515,598,736 recoveries [3]

SARS-CoV-2 is an enveloped virus with a positive-strand RNA genome and four structural proteins: spike (S), envelope (E), membrane (M), and nucleocapsid (N). Since its first appearance in 2019, variants of SARS-CoV-2 have continued to emerge. Among the most common variants, the Omicron variant is responsible for the largest surge of COVID cases since 2019 [4]. However, the Omicron variant has caused significantly less morbidity and mortality. Variants of SARS-CoV-2 preferentially infect the airway mucosa [4]. As such, respiratory symptoms such as fever, cough, and shortness of breath are among the most common manifestations of the infection [1,4,5]. However, many factors affect the prognosis and severity of the disease [4,5].

It has been found that the spike protein of SARS-CoV-2 has a broad tropism for mammalian angiotensin-converting enzyme 2 (ACE2). This property of the spike protein allows SARS-CoV-2 to infect tissues that express ACE2 receptors [5,6]. ACE2 receptors are found on type II alveolar cells (AT2) [7,8,9,10], myocardial cells [7], esophagus epithelial cells [7,8,9,10], neurons and glia [11], tongue epithelial cells [12], and the oral cavity [12]. Infections of these tissues are consistent with COVID-19 symptoms.

SARS-CoV-2 virus entry into cells occurs through the attachment of ACE2 receptors and leads to a sequential cascade of events [7,12,13,14]. After the attachment of the S protein onto ACE2, a type II transmembrane serine protease (TMPRSS2) activates ACE2. The binding of ACE2 receptors and subsequent gene activation of TMPRSS2 leads to downstream activation of spike protein and facilitates viral entry into the host cell via receptor-mediated endocytosis [12,13,14]. TMPRSS2 gene expression is promoted via androgen receptors and TMPRSS2 gene expression increases when exposed to androgens [13,14]. To date, no other regulatory elements (beyond androgen receptors) have been identified to increase TMPRSS2 gene expression [14]. A robust association between androgens and TMPRSS2 has been analyzed in the literature [13,14]. This correlation elucidates the greater susceptibility to COVID-19 in males, as males typically maintain higher levels of androgens. This association clarifies the less symptomatic disease in children with overall low expressions of androgen receptors [7,13,14,15,16,17].

Most people infected with SARS-CoV-2 experience mild respiratory symptoms such as dry cough, anosmia, and nasal congestion [15,16,17]. Extrapulmonary symptoms have also been heavily reported. Extrapulmonary symptoms include myocarditis, acute kidney injury, gastrointestinal damage, liver damage, thrombotic events, endocrine dysfunction, and neurological complications [18].

Additionally, newly emerging dermatological manifestations have been observed in COVID-19 patients. Such findings are consistent with a recent discovery that keratinocytes express ACE2 receptors, making the skin a potential target for SARS-CoV-2 infection [19]. Other possible mechanisms for COVID-19-related cutaneous manifestations include hypersensitivity of the immune system in response to SARS-CoV-2 RNA, cytokine release, microthrombi formation, and vasculitis development [20].

To better comprehend COVID-19-related cutaneous manifestations, the International League of Dermatological Societies (ILDS) collaborated with the American Academy of Dermatology (AAD) to create an online registry of data collection about COVID-related dermatological conditions. As of March 2021, the AAD/ILDS COVID-19 Dermatology Registry has documented 1875 entries from 52 countries [21]. The most prevalent dermatological conditions related to COVID-19 include (1) exanthematous (morbilliform) rash (22%), (2) pernio-like acral lesions (18%), (3) urticaria (16%), (4) varicella-like eruption (11%), (5) papulosquamous rash (9.9%), and (6) retiform purpura (6.4%) [21,22]. Additionally, rare cases of multisystem inflammatory syndrome in children (MIS-C) have been attributed to COVID-19 infections [23,24,25]. MIS-C patients may present with mucocutaneous manifestations and other systemic symptoms such as fever and gastrointestinal symptoms. Clinical symptoms of MIS-C can present similarly to Kawasaki Disease. However, MIS-C is differentiated by several distinct clinical patterns including the average age of onset, cardiovascular sequelae, and laboratory tests [26,27].

The COVID-19 pandemic has led to increased personal protective equipment (PPE) usage among the general population and healthcare workers. PPE usage has been shown to trigger dermatological conditions such as facial dermatoses, contact dermatitis, acne, and friction dermatitis [28,29,30,31,32]. Furthermore, the long-term usage of protective clothing and contacting disinfectants can disrupt the skin barrier, increasing the risk for infections and autoimmune conditions [33].

Efforts to reduce the spread of SARS-CoV-2 led to the accelerated development of several COVID-19 vaccines. At this time, mRNA and DNA-based COVID-19 vaccinations are available worldwide. In the United States, COVID-19 mRNA vaccinations have been widely available to the public through expedited FDA approval, conditional marketing approval and emergency use authorization pathways [34]. COVID-19 vaccination excipients vary according to the vaccine manufacturer. Polyethylene glycol (PEG) is a vaccine excipient that has been used as an ingredient in Pfizer and Moderna mRNA vaccines. Contrastingly, Polysorbate 80 is a vaccine excipient in the Johnson & Johnson COVID-19 vaccine [35]. Cutaneous adverse events to these vaccine excipients have been reported, including allergic reactions at the injection site (COVID arm), chilblain-like lesion, urticaria, and anaphylaxis [36,37,38].

The COVID-19 pandemic has disrupted society through government lockdowns, quarantine measures, isolation, fear of infection, the stress of employment, changes in educational delivery, and bereavement of loved ones [39,40,41]. Moreover, these unique psychosocial stressors have been linked to exacerbations of pre-existing psoriasis, eczema, telogen effluvium, and atopic dermatitis [42,43,44,45].

**Figure 1:**
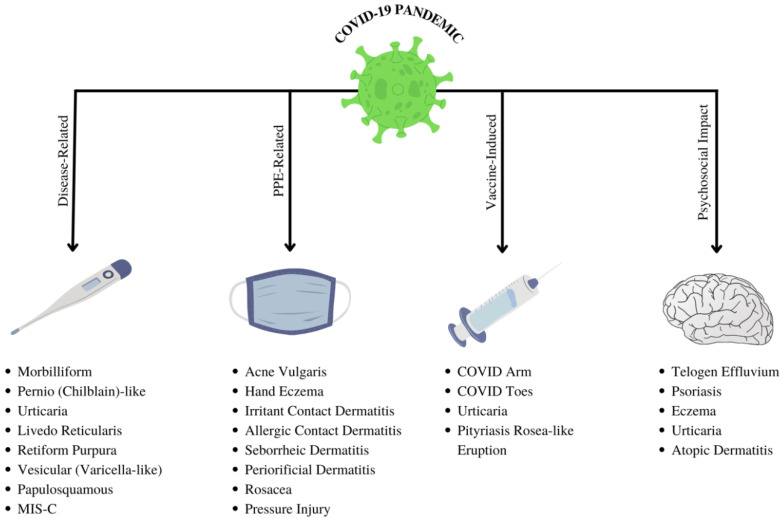
Overview of Cutaneous Manifestations Associated with the COVID-19 Pandemic. MIS-C, Multisystem Inflammatory Syndrome in Children; COVID, Coronavirus Disease.

This comprehensive literature review is the first broad-spectrum COVID-19 examination of dermatological manifestations associated with the COVID-19 pandemic: infections, vaccinations, PPE, and pandemic-related psychosocial factors. Our primary objective was to collate and categorize dermatological conditions and treatments associated with the COVID-19 pandemic according to the four pandemic-related domains outlined in Figure 1. The findings reflect a detailed culmination of scientific literature and data. This review was designed to provide a practical framework for clinicians and researchers, with emphasis on etiologies, risk factors, prevention and management for pandemic-related cutaneous manifestations.

## 2. Methods

A literature search was conducted using keywords related to the cutaneous manifestations during the COVID-19 pandemic on PubMed and MEDLINE. The literature search was designed to extract research articles that discuss dermatological conditions associated with the COVID-19 pandemic: infection, vaccines, personal protective equipment (PPE), and psychosocial factors. Key terms used in PubMed and MEDLINE included: “Dermatology” OR “Skin” OR “Eczema” OR “Psoriasis” OR “Exanthem Rash” OR “Dermatitis” OR “Pityriasis Rosea” OR “Livedo Reticularis” OR “Urticaria” OR “Telogen Effluvium” OR “Blue Toes” OR “COVID ARM” OR “COVID Vaccine” OR “Stress” OR “Depression” OR “Anxiety” OR “PPE” AND (COVID OR COVID-19 OR SARS-CoV-2). Publication dates were set from December 2019 to August 2021. Articles written in a language other than English were excluded. Abstracts, animal research, and pending articles were excluded from the literature search. The search retrieved 2199 articles initially screened by two reviewers (PO and WH). All articles were screened based on title and abstract. Articles were initially included if it contained any of the utilized key terms in its title or abstract. Following the preliminary screening, pertinent meta-analyses, systematic reviews, prospective studies, retrospective studies, topic reviews, case series, and case reports were included. No randomized controlled trials (RCTs) were identified (within the scope of the literature search) at the time of the literature search.

## 3. Results

The COVID-19 pandemic has triggered a significant range of dermatologic sequela. However, a direct causal relationship has not yet been fully established. Several hypotheses have been proposed to explain the underlying pathogenesis concerning SARS-CoV-2 infection. One prominent mechanism describes viral spike protein invasion into the dermis by binding to angiotensin-converting enzyme 2 (ACE2) receptors in keratinocytes, with a secondary immunological response. The literature revealed a vast array of dermatoses associated with the pandemic: infection, vaccinations, personal protective equipment (PPE), and psychosocial factors. Following vaccination, allergic reactions, anaphylaxis, and other adverse events have been reported. Prolonged PPE usage has been shown to cause contact dermatitis, acne, periorificial dermatitis, and pressure injuries. Pandemic-related psychosocial factors include impacts of quarantine, financial uncertainty, restricted measures, and significant life changes. The pandemic has caused a marked increase in stress-responsive dermatological conditions such as telogen effluvium, psoriasis, eczema, urticaria, and atopic dermatitis.

## 4. COVID-19 Specific Dermatological Manifestations

### 4.1. Exanthematous (Morbilliform) Rash

Exanthematous rashes exist as a broad category, but are classically described as viral exanthems or drug-induced type IV hypersensitivity reactions [46]. Morbilliform rash is the most common cutaneous reaction associated with COVID-19 infection [23,47,48]. It accounts for 11% to 47% of all dermatological cases related to COVID-19 (Table 1) [23,48]. Additionally, retrospective studies and case series reported a higher rate of hospitalization (45% to 80%) in COVID-19 patients with morbilliform eruptions compared to COVID-19 patients without the rash [23,48,49,50,51].

**Figure 2:**
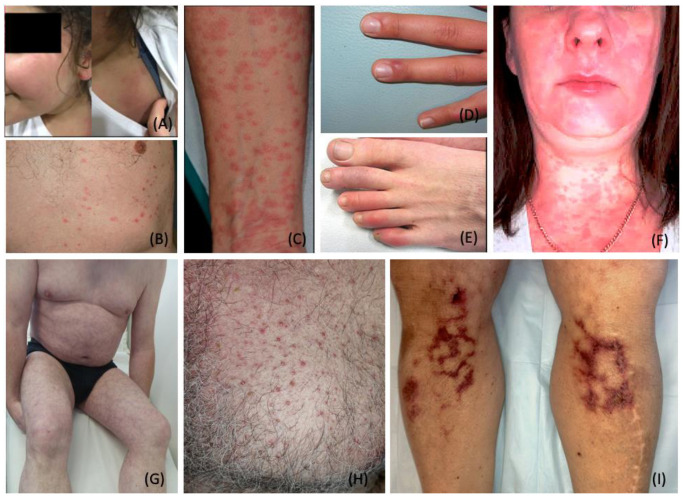
Cutaneous Manifestations Related to COVID-19 Infection. (**A**). Maculopapular rash observed on the face and shoulder of an 11-year-old child; (**B**). Varicella-like crusted papular lesions of the trunk; (**C**). Maculopapular lesions coalesced into small plaques on the anterior forearm; (**D**,**E**). Chilblains-like erythema of the fingers (**D**) and toes (**E**); (**F**). Urticarial erythematous eruption of the face, neck, and upper chest; note: no mild angioedema on the lower lip, due to excess interstitial fluid in the dermis and subcutaneous tissue; (**G**). Livedo reticularis: a symmetric regular lace-like network of the trunk and extremities (**H**). Monomorphic vesicles and pustules of the chest; (**I**). Retiform purpura of the lower extremities. Photo credit is acknowledged to the following original reports: Burcu Bursal Duramaz [140], Raffaele Gianotti [141], Michel Verheyden [94], Khalid Hassan [87], Xavier Bosch-Amate [135].

The temporal association between the onset of morbilliform rash and other COVID-19-related symptoms varies among patients. Morbilliform rashes tend to occur concurrently or after the emergence of other COVID-19-related symptoms [23]. Morbilliform eruptions are associated with moderate to severe COVID-19 symptoms [23,49,52,53]. Among COVID-19 patients, the most common anatomical locations for morbilliform eruptions include the chest, abdomen, back, arms, and legs (Figure 2A–C) [23,50,52,54].

Histological examinations of skin biopsies from patients with morbilliform rash and concomitant SARS-CoV-2 infection reveal spongiosis and perivascular inflammatory infiltrates. Such lesions may present with edematous thrombosed vessels surrounded by neutrophils and eosinophils [13]. However, the SARS-CoV-2 spike protein has not been detected in biopsy samples [13,55,56]. These findings suggest morbilliform eruptions are not directly caused by the virus but rather may be secondary to the inflammatory response of the infection [55].

COVID-19-induced morbilliform exanthem treatment protocols are limited to those described in case reports. Most cases of morbilliform eruptions are self-resolving and do not require treatment. However, in severe symptomatic cases, treatment may be necessary for symptomatic relief (i.e., pruritus). For example, one case study reported a 58-year-old male who experienced complete resolution upon treatment with topical triamcinolone 0.1% cream [57]. Another case reported positive treatment outcomes with the administration of topical corticosteroids (type unspecified) and oral antihistamines (Table 1) [54].

### 4.2. Pernio (Chilblain)-like Acral Lesions

Chilblains-like lesions typically present as violaceous, erythematous to purpuric plaques with or without edema. While chilblains lesions can present in multiple body regions, these lesions are typically seen on the fingers or toes (Figure 2D,E) [58,59]. Chilblains lesions were relatively rare before the COVID-19 pandemic, with approximately nine to ten cases reported annually between 2000 and 2011 [60]. However, since the onset of the COVID-19 pandemic, cases of chilblains-like rashes have been on the rise. Multiple studies reported that chilblains-like eruptions comprised approximately 19% to 38% of all dermatological manifestations related to COVID-19 infections (Table 1) [23,48,61].

The term “chilblains-like” lesion was coined to delineate skin lesions that mimicked primary chilblains in patients with COVID-19 infection. Primary chilblains develop after exposure to low temperatures. In contrast, many COVID-19 patients with “chilblains-like” lesions had no cold exposure or history of similar rash [62]. Several mechanisms have been proposed to describe the pathogenesis of chilblains-like rashes. One prominent hypothesis suggests that during the active infection phase, Type 1 interferon promotes the production of cryofibrinogen. Cryofibrinogen is an acute phase reactant that can induce perniosis at acral sites [60].

Histopathological examinations of COVID-19 chilblains-like lesions reveal the presence of superficial and deep lymphocytic inflammatory infiltrates in a lichenoid, perivascular, and perieccrine pattern [13,60,63,64,65]. Additionally, SARS-CoV-2 spike proteins were detected in the endothelium of blood vessels in some samples but absent in others [66,67,68]. This discrepancy likely demonstrates complex relationships with the SARS-CoV-2 spike protein and the resulting inflammatory cascade. These findings indicate a direct causal relationship between SARS-CoV-2 and chilblains-like eruptions [69,70].

Among COVID-19 patients, chilblains-like rashes typically spontaneously resolve within two to eight weeks [23,24]. However, underlying persistent inflammation may have contributed to prolonged cases lasting over six months [71]. Patients should avoid cold exposure to prevent flare-ups. In refractory cases, corticosteroids, or calcium channel blockers (e.g., nifedipine) can provide therapeutic relief (Table 1) [72,73].

### 4.3. Urticaria

Urticaria is characterized by well-circumscribed, edematous, raised, pruritic, and erythematous plaques (Figure 2F) [74]. Causes of urticaria include but are not limited to allergens, insects, medications, and infections [75]. In addition, viruses such as rhinovirus, rotavirus, hepatitis A, hepatitis B, and Epstein-Barr virus (EBV) are known to trigger urticarial eruptions [75]. The pathogenesis of urticaria involves the degranulation of mast cells or basophils and the release of histamine in the upper dermis. Histamine released in the deeper dermis increases vasculature permeability, leading to angioedema [76].

SARS-CoV-2 infection appears to induce urticaria [48,77,78,79]. Several international studies report urticaria among 8–19% of skin lesions related to COVID-19 (Table 1) [23,48,80]. Several studies proposed associations between the severity of urticarial eruptions and COVID-19 infections, but the data are mixed. Casas et al. reported high morbidity and mortality in a subset of their cohort (2%) with urticaria. However, Dastoli et al. demonstrated that COVID-19 patients with urticaria have a better prognosis, potentially due to eosinophilia [81,82]. Currently, the protective mechanism of eosinophilia against COVID-19 is unknown [83,84].

Histological findings of urticarial eruptions related to COVID-19 are nonspecific but consistent with viral urticarial exanthems. Such findings consist of papillary dermal edema and mild perivascular lymphocytic infiltrate with some eosinophils, though neutrophilia predominate in early urticaria [13]. One case that appeared clinically consistent with urticaria had histopathologic findings comparable to erythema multiforme, with slight vacuolar interface dermatitis and occasional necrotic basal keratinocytes [13]. Given the uncertain clinicopathological mechanism, biopsies of future urticarial eruptions in the setting of COVID-19 should be considered for further evaluation [85].

Urticarial eruptions associated with SARS-CoV-2 infection have variable presentations. One case reported a 27-year-old woman who developed angioedema and urticarial rash one week after confirmed diagnosis of SARS-CoV-2 infection [86]. The urticaria persisted for 12 weeks despite treatment with cetirizine 20 mg twice daily [86]. Other studies have reported urticaria as the initial or only clinical sign in several patients with COVID-19 infection [48,87,88,89]. As such, physicians should recognize symptoms of urticaria and fever in patients to limit the spread of COVID-19 [90].

The primary treatment of urticaria in patients with COVID-19 includes second-generation oral antihistamines. One case report depicts the resolution of a unilateral upper-extremity urticarial rash within 24 h after treatment with oral antihistamines and topical corticosteroids [91]. Other investigations have proposed low-dose systemic corticosteroids as a viable treatment option, though more clinical data are needed (Table 1) [92].

### 4.4. Livedo Reticularis

Livedo reticularis (LR) is distinguished as a transient or persistent vascular, violaceous, net-like skin discoloration (Figure 2G). LR occurs secondary to physiological or pathological reduction in blood flow to the skin [93]. According to the AAD’s Dermatology Registry, livedo reticularis comprised 5.3% of confirmed COVID-19 cases with cutaneous manifestations (Table 1) [23]. Histologic findings of livedo reticularis in a patient with confirmed COVID-19 revealed pauci-inflammatory thrombotic vasculopathy [23]. However, most reported cases of livedo reticularis in COVID-19 patients were mild, transient, and without thromboembolic complications [23].

Limited reports depict the treatment of LR in patients with COVID-19. One case study described a patient who developed LR on the trunk and bilateral proximal upper extremities following COVID-19 infection. Therapy with acetaminophen, heparin, hydroxychloroquine, and oxygen resolved LR in the patient [94]. In contrast, other cases of COVID-19-related LR have resolved spontaneously (Table 1) [95]. Additional research is needed to elucidate the best management strategies for these patients.

### 4.5. Livedo Racemosa/Retiform Purpura

Livedo racemosa and retiform purpura are vaso-occlusive lesions of the superficial microvasculature. These lesions are associated with elevations in d-dimer and disseminated intravascular coagulopathy (DIC), as seen in those with severe COVID-19 infections [96,97]. Rather than the fine net-like pattern of livedo reticularis [98]. In contrast to the transient nature of livedo reticularis, livedo racemosa presents as a violaceous mottling of the skin in a disorganized pattern or broken circular segments. Livedo racemosa is pathologic, persistent, and associated with increased severity of ischemia. Differentiation between the two lesions is often distinguished based on pattern, histological examination, and location on the body. In terms of anatomical location, livedo racemosa is more commonly found on the trunk, limbs, and buttocks [93]. Upon biopsy with immunochemical staining, these vaso-occlusive lesions demonstrate evidence of complement activation with immunoglobulins and microthrombi within the vasculature of the superficial and mid-dermis [96].

According to the AAD Data Registry, retiform purpura and livedo racemosa presented in 6.4% and 2.3% of COVID-19 patients with dermatological conditions, respectively (Table 1) [23]. These lesions correlate with increased severity of COVID-19 infection and mortality. One hundred percent of patients with documented retiform purpura were hospitalized, and 82% developed acute respiratory distress syndrome (ARDS) [23,96]. These vaso-occlusive conditions are consistent with the highest mortality rate of all cutaneous manifestations at 18.2%, with urticaria-like lesions ranking the lowest at 2.2% [99]. Management incorporates treatment to address underlying pathology, anticoagulation, a biopsy of skin lesions, and wound care [100].

Purpuric pressure ulcers have been observed during the COVID-19 pandemic on the back, buttocks, and other pressure-dependent locations [97]. In a case series by Chand et al., a biopsy of lesions presented with no evidence of thrombotic vasculopathy. The mechanism of necrosis may have been due to pressure occlusion rather than inflammation or thrombosis. Additionally, those with purpuric pressure ulcers appeared with no laboratory evidence of DIC [96,97].

### 4.6. Vesicular (Varicella-like) Eruptions

Vesicular eruptions are among the most common COVID-19-associated dermatological manifestations. International case series and retrospective cohort studies identified varicella-like rashes in 9% to 13% of COVID-19 patients with cutaneous eruptions (Table 1) [23,48,101,102]. In relation to other specific COVID-19 symptoms, the onset of varicella-like rashes varies among patients. The majority of lesions appear three days after systemic symptoms such as fever, cough, and fatigue [48,102]. On average, resolution occurs by day eight without residual scarring [23,48,102]. However, a minority of patients developed vesicular lesions prior to the onset of COVID-19 symptoms [23,48].

Varicella-like eruptions are classified into two morphological patterns: (1) diffuse (more common) and (2) localized [103]. The diffuse pattern consists of small papules, vesicles, and pustules of varying sizes. These lesions appear at different stages simultaneously, starting on the trunk (most commonly) with further spreading to palms, soles, and other corporal areas [103]. The localized monomorphic pattern typically involves the trunk and back (Figure 2H) [103]. In contrast to the diffuse pattern, varicella-like lesions associated with the localized pattern are monomorphic and appear at the same stage of evolution. These lesions affect one central area involving the chest, upper abdomen, or back [103].

Similar to varicella-exanthems, varicella-like eruptions associated with COVID-19 can be diffuse or localized and also predominantly involve the trunk [96,102]. Unlike true varicella exanthems, most cases of varicella-like eruptions related to COVID-19 have been documented as non- or mildly pruritic [96,102].

Histological findings of varicella-like lesions vary between patients. Most studies report histological patterns consistent with viral exanthems, such as vacuolar degeneration of the basal layer with multinucleate, hyperchromatic keratinocytes, and dyskeratotic cells [102,104]. Contrastingly, other studies have documented the presence of non-ballooning acantholysis with eosinophilic dyskeratosis [105]. Despite the histological variations, the consistent clinical morphology and presentation of these lesions assists in identifying patients with COVID-19 infection [103].

The data surrounding the treatment of varicella-like eruptions in the literature is limited. A case series (*n* = 22) presented by Marzano et al. demonstrated lesions spontaneously resolved within eight days of systemic symptoms [103]. At this time, watchful waiting is recommended as an effective treatment (Table 1).

### 4.7. Papulosquamous Rashes and Pityriasis Rosea

Pityriasis rosea (PR) manifests as typical papulosquamous eruptions characterized by ovoid and raised scaly patches on the body [96]. Classically, pityriasis rosea presents as a solitary lesion (herald patch) and progresses along the Langer lines as a generalized rash over the trunk and limbs [106]. Papulosquamous eruptions comprised 9.9% of cutaneous manifestations related to COVID-19 in the AAD Dermatology Registry [23]. Additional studies have documented an increased incidence of PR-like lesions in association with SARS-CoV-2 infection [107,108,109,110,111].

However, cases of PR in patients with COVID-19 infection may not present with the classic herald patch. In one case report, a 49-year-old female patient with COVID-19 pneumonia developed a papulosquamous rash resembling pityriasis rosea three days prior to the development of COVID-19-specific symptoms. Supportive treatment improved her respiratory and dermatological symptoms on day 5 of hospitalization [112]. In another case report, Sanchez et al. documented an atypical digitate papulosquamous variant in an elderly patient (age not specified) with a confirmed diagnosis of COVID-19. The lesions were clinically similar to pityriasis rosea, however, without the presence of a herald patch [113]. The rash resolved within one week. However, the patient expired due to an infection-related sequelae.

The specific etiology of papulosquamous eruptions in patients with COVID-19 remains unknown. It is postulated that increased inflammatory cytokines may contribute to rash development [113]. Other potential causes include viral reactivation of Human Herpes Virus-6 and 7 (HHV-6 and 7) [114], direct SARS-CoV-2 spike protein infection of the endothelium of dermal blood vessels [115], or viral EBV reactivation [113,114].

Histological findings of papulosquamous eruptions are characterized by spongiosis with focal parakeratosis in the epidermis with aggregates of lymphocytes and Langerhans cells [23,113,116]. Treatment for papulosquamous eruptions is unnecessary and warrants watchful waiting (Table 1). For patients who present with papulosquamous eruptions, it is recommended to test for COVID-19, EBV, and HHV-6/7 [96]. It remains uncertain whether a direct association exists between COVID-19 infection and the development of papulosquamous eruptions. Further research is needed to extrapolate a potential pathomechanism between SARS-CoV-2 infection and such skin lesions.

### 4.8. Multisystem Inflammatory Syndrome in Children

Children infected with SARS-CoV-2 are often asymptomatic or exhibit mild symptoms [117,118]. Although quite rare, a small percentage of MIS-C cases in children with COVID-19 required hospitalization, secondary to shock and multiorgan system failure. These children had symptoms comparable to toxic shock syndrome (TSS) and Kawasaki disease (KD) [119,120,121,122,123]. Consequently, the CDC released an official report and used the term Multisystem Inflammatory Syndrome in Children (MIS-C) to define this novel condition. In the United States, the CDC and WHO diagnostic guidelines for this condition require the presence of all of the following:

1. Fever
2. Inflammatory markers
3. Failure or involvement of two organ systems
4. Evidence of COVID-19 infection or exposure [117,124,125].

Most children who met the criteria for MIS-C presented with a multitude of mucocutaneous findings [126]. Thus, it is vital for dermatologists to be aware of this condition and its dermatological manifestations and potential sequelae. The mucocutaneous findings of MIS-C include conjunctival injection, palmoplantar erythema, lip hyperemia, periorbital erythema and edema, strawberry tongue, and malar erythema [127,128]. Similar to the mucocutaneous manifestations of Kawasaki disease, defining features of MIS-C are outlined in Table 2 to help differentiate between MIS-C and KD.

Patients diagnosed with MIS-C require immunomodulatory treatment and multidisciplinary care. In the United States, the CDC published management guidelines that specify IVIG and glucocorticoids as first-line treatments. The interleukin-1 (IL-1) receptor antagonist anakinra is recommended for refractory cases of MIS-C. Low-dose aspirin, enoxaparin, and tocilizumab can also be prescribed in certain cases (Table 1) [129].

**Table 1:** Characteristics of Common COVID-19-related Dermatologic Manifestations.

Rash	Relative Frequency ^a^	COVID Severity ^b^	Reported Treatments
Exanthematous (Morbilliform)	11–45% [48,130]	Moderate-severe	–Topical corticosteroids [54,57] – Oral antihistamines [57,131] – Hydroxychloroquine [131] –Low molecular heparin (if suspected coagulopathy) [131]
Pernio (Chilblains-like)	18–53% [48,132]	Mild	–Avoid cold temperatures and vasoconstrictive agents –Watchful waiting [133,134] –Corticosteroids or nifedipine for refractory cases [133,134]
Urticaria	8.1–19% [23,48,80]	Moderate	–Second-generation antihistamines [91] –Topical corticosteroids [91] –Low-dose systemic corticosteroids [92]
Vesicular (Varicella-like)	9–19% [48,101]	Moderate	– Watchful waiting [102]
Papulosquamous	9.9%	Moderate	–Not reported
Retiform Purpura	6.4%	Severe	– Anticoagulation [135,136]
Livedoid Reaction	5.3%	Mild	–Watchful waiting [95]
MIS-C ^c^	0.6%	Severe	–Multidisciplinary approach –IVIG –Anakinra –Low dose aspirin –Enoxaparin –Tocilizumab [129]

^a^ Relative frequency of rash in laboratory-confirmed COVID-19 compared to other dermatologic manifestations of COVID-19. Ranges were adapted from both Freeman et al. [23] and from the reports cited in the column. ^b^ Severity is based on the percentage of patients with each condition who were hospitalized with COVID-19 [23]. ^c^ MIS-C, Multisystem Inflammatory Syndrome in children.

**Table 2:** Differentiation of Multisystem Inflammatory Syndrome in Children (MIS-C) and Kawasaki Disease (KD).

	MIS-C	KD
Epidemiology	Non-Hispanic blacks are at higher risk [137,138]	Asians are at higher risk
Age of onset	8–12 years [139]	<5 years
Fever	Fever >24 h	Fever > 5 days
Cardiovascular Abnormalities	Myocarditis/myocardial dysfunction (left ventricular dysfunction)	Coronary artery abnormalities such as aneurysms are more common
Platelet Count	Thrombocytopenia	Thrombocytosis

## 5. Dermatological Conditions Associated with Personal Protective Equipment and Hygiene Products

**Figure 3:**
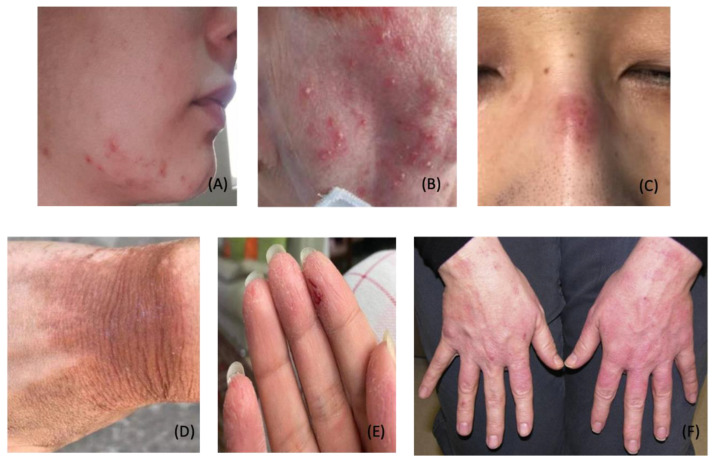
Dermatological Conditions Related to PPE and Hygiene Products. (**A**). Acne vulgaris; (**B**). Rosacea; (**C**). Pressure injury; (**D**,**E**). Irritant contact dermatitis; (**F**). Hand eczema; Photo credit is acknowledged to the following original reports: Mohammed Shanshal [154], Anca E. Chiriac [155], Z Q Yin [156], Mohammed Shanshal [154], Chandler W. Rundle [157].

The COVID-19 pandemic has led to increased personal protective equipment (PPE) use among the general population and healthcare workers. Personal protective equipment protects the wearer from the transfer of disease through air, fluids, or direct contact. Pandemic healthcare workers have developed dermatological conditions due to prolonged use of personal protective equipment (PPE). Increased use of personal protective equipment (e.g., goggles, gloves, N95 face masks, and alcohol-based hand rubs) has led to a multitude of dermatological conditions: acne, periorificial dermatitis, papulopustular rosacea, pressure injury, irritant contact dermatitis, allergic contact dermatitis, hand eczema, and seborrheic dermatitis [28,29,30,31,32]. (Figure 3A–E) Long-term usage of protective clothing and contact disinfectants may disrupt the skin barrier and increase the risk of infection and autoimmune conditions [33]. Dermatoses related to the prolonged wearing of PPE can be managed with prophylactic hygiene measures, conservative treatment, and pharmacological therapies (Table 3).

**Table 3:** PPE-related Skin Conditions and Treatment Approaches.

	Description	Prophylactic Approach	Treatment Approach
Acne	Inflammation of pilosebaceous glands, likely worsened by heat and humidity from face mask microenvironment [29]	–Use salicylic acid or benzoyl peroxide wash for daily facial cleansing –Use non-comedogenic products –Use a cotton mask when possible [142] –Take a 15-min mask break every 2 h [143]	–**Mild:** Salicylic acid or benzoyl peroxide wash daily; topical retinoid; topical antibiotics –**Moderate:** Follow recommendations for mild, add oral antibiotics (i.e., doxycycline); consider oral spironolactone or combined oral contraceptives for female patients –**Severe:** Consider isotretinoin [144]
Periorificial Dermatitis	Inflammatory papulopustular condition with underlying erythema surrounding eyes, nose and/or mouth, likely worsened by face mask microenvironment [29]	–Avoid topical steroids –Use gentle cleansers –Take a 15-min mask break every 2 h [143]	–**Mild:** Topical calcineurin inhibitors, erythromycin, and/or metronidazole [145] –**Moderate/Severe:** Oral tetracyclines [146]
Papulopustular Rosacea	Facial erythema with telangiectasias, typically overlying the malar and nasal bridge regions, is likely worsened by the face mask microenvironment [29]	–Gentle skin cleansing –Use gentle moisturizers daily –Avoid potential triggers (i.e., spicy food, alcohol, direct sunlight, acute physiologic stressors) [147]	–**Mild:** Topical azelaic acid, topical ivermectin, or metronidazole; consider sodium sulfacetamide wash –**Moderate:** Consider oral doxycycline –**Severe:** Consider isotretinoin [148] –**Erythema:** Topical brimonidine or oxymetazoline [144]
Pressure Injury	Damage to skin and soft tissue due to continuous pressure and/or shear force; may present as erythema or ulcerated skin, often over the bridge of the nose, behind ears, or on cheeks [149]	–Ensure appropriate mask fit –Topical application foam or hydrocolloid dressing at contact points [150,151]	–Use moisturizers, skin sealants, cyanoacrylate –Use foam or hydrocolloid dressing at contact points [149]
Contact Dermatitis (Irritant)	Inflammation is caused by direct physical or chemical insult, often behind the ears, on cheeks, or over the nasal bridge [29]	–Avoid allergens –Use a cotton mask when possible –Use foam or hydrocolloid dressing at contact points [143,150,151] –Take 15-min mask break every 2 h [143,151]	– Topical steroids or calcineurin inhibitors [144]
Contact Dermatitis (Allergic)	Localized and well-demarcated type IV hypersensitivity reaction; common triggers found in PPE include formaldehyde, dibromodicyanobutane, thurium, and metal wire [29]		–**Localized:** Topical steroids or calcineurin inhibitors –**Widespread:** Systemic corticosteroids and other immunosuppressive therapies [144]
Hand Eczema	Itchiness, dryness, and redness of the hands due to excessive hand washing, disinfectants, and glove use [144]	–Minimize use of hot water –Frequent use of hypoallergenic moisturizer –Avoid allergens and/or irritants [152,153]	–Nighttime petroleum-based emollient with cotton gloves –Short course topical corticosteroids [144]
Seborrheic Dermatitis	Superficial fungal infection, often affecting ears, nasolabial folds, eyebrows, and scalp; likely worsened by face mask microenvironment [29]	–Gentle skin cleansing –Frequent moisturizer use [144]	–Topical ketoconazole shampoo, body wash, or cream –For itching and erythema, mild topical corticosteroids or calcineurin inhibitors can be used [144]

Abbreviations: PPE, personal protective equipment.

## 6. COVID-19 Vaccine-Induced Dermatological Manifestations

COVID-19 vaccinations were developed to reduce the spread and severity of SARS-CoV-2 infection. At this time, mRNA and DNA-based COVID-19 vaccinations are available worldwide. In the United States, COVID-19 mRNA vaccinations have been widely available to the public through FDA approval, conditional marketing approval, and emergency use authorization pathways [158,159,160].

In the United States, adverse vaccine reactions are documented in a federal vaccine surveillance registry known as Vaccine Adverse Events Reporting System (VAERS). As a passive reporting system, VAERS data has been estimated to be largely underreported with incomplete data [161]. The emergence of mass worldwide SARS-CoV-2 vaccination has resulted in an increased number of diverse adverse vaccine reactions. The greatest absolute risks related to COVID-19 vaccinations include, but are not limited to, allergic, constitutional, cardiovascular, dermatological, gastrointestinal, neurological, and localized pain.

A research study compared the documented adverse events of the COVID-19 vaccines administered between 2020 and 2021 to those of the influenza vaccines administered within the same year in the United States and Europe. All individuals who received either the COVID-19 or influenza vaccines were 18 years and older. A similar number of people received either COVID-19 vaccines (*n* = 451 million) or the influenza vaccines (*n* = 437 million). Results of the study revealed that more serious adverse events were associated with the COVID-19 vaccines in comparison to the influenza vaccines. Greater adverse events were quantified as allergic reactions, arrhythmias, general cardiovascular events, coagulation, hemorrhage, constitutional, ocular, sexual organ, and gastrointestinal reactions, with a greater relative risk for thromboembolic events [161].

Anaphylactic urticaria and other dermatological manifestations currently comprise a smaller percentage of COVID-19 vaccination adverse events [161,162]. Anaphylaxis is a serious and life-threatening complication associated with COVID-19 vaccines. Anaphylaxis presents as an acute, life-threatening hypersensitivity reaction that affects multiple organ systems. Symptoms range from mild wheals, pruritus, and urticaria to severe respiratory symptoms [163]. An increasing number of cases of anaphylaxis continues to be reported worldwide, and should be adequately addressed by regulatory authorities and pharmaceutical manufacturers [164,165].

The Pfizer and Moderna mRNA vaccines are packaged in lipid nanoparticles (LPNs) that contain polyethylene glycol (PEG), proteins, and other lipids [166,167]. PEG has been suggested as a potential allergen in vaccines [166,167]. Multiple cases of severe allergies to PEG have been reported [168,169,170,171]. Due to low awareness of PEG allergy, many cases have been misdiagnosed as idiopathic anaphylactic reactions [170,171,172].

The proposed mechanisms of anaphylaxis to PEG are defined as IgE-mediated or complement-mediated [169,173,174]. Currently, no approved allergy testing exists for PEG. Although skin testing has been used to investigate reactions to PEG, severe allergic reactions have been reported after the intradermal injection of this agent [171]. The underlying immunological mechanism of allergic reactions to the COVID-19 vaccines remains poorly understood. More research is needed to devise appropriate, cost-effective pre-screening methods for vulnerable populations.

**Figure 4:**
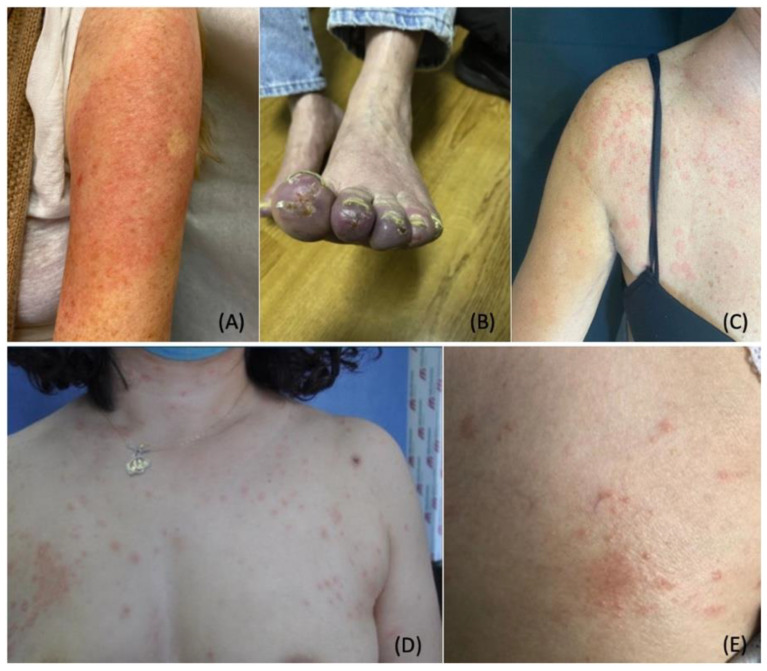
Cutaneous Manifestations Associated with COVID-19 Vaccination. (**A**). Pruritic and erythematous rash was observed on the left arm of a 74-year-old woman eight days following the inoculation of the Moderna vaccine. (**B**). Pernio/chilblains-like lesion of the toes observed in a 76-year-old man one week after receiving the second dose of the Moderna vaccine. (**C**). Urticarial wheals on the trunk of a 48-year-old woman three hours after receiving the second dose of the Oxford-AstraZeneca vaccine. (**D**,**E**). Oval salmon-colored plaques on the trunk and herald patch on the right breast of a 45-year-old woman four days after receiving the first dose of the CoronaVac vaccine. Photo credit is acknowledged to the following original reports: Nancy Wei [180], John M. Kelso [181], Enes Yağız Akdaş [184], Martina Burlando [185].

Another emerging complication of COVID-19 vaccines includes COVID arm. (Figure 4A) “COVID arm” is a term coined to describe a post-vaccination reaction characterized by an erythematous rash surrounding the injection site [175]. Multiple case series revealed the majority of COVID arm cases develop after the first dose of the Moderna vaccine with a median onset of eight days post-vaccination [38,175,176,177]. Histological findings of these skin lesions revealed a predominance of CD4+ helper T cells with variable eosinophils, consistent with delayed-type hypersensitivity reactions (DTHR) [38,175,176].

As discussed earlier, Moderna and Pfizer vaccines contain excipients such as polyethylene glycol, which is proposed to elicit DTHR [178]. In the United States, Pfizer vaccines have been administered in similar amounts to the Moderna vaccine [179]. However, the incidence of COVID arm after the administration of the Pfizer vaccine is significantly lower compared to the Moderna vaccine [175,176,177,180]. Underreporting of vaccine reactions in the United States VAERS may account for the difference in numbers [161]. COVID arm symptoms resolve spontaneously after three to eight days [177]. Treatment with topical steroids, ice, or oral antihistamines can be implemented for expedited recovery and symptom relief [177,180].

Chilblains-like rash (“COVID toes”) is another cutaneous reaction that has been associated with the Pfizer and Moderna vaccines. (Figure 4B) COVID toes typically appear four to seven days post-vaccination [38,181]. This localized reaction is hypothesized to manifest as an immune response to the vaccination. On histological examination, interstitial lymphocytic inflammatory infiltrates have been observed on punch biopsy [181,182]. COVID toes have been documented to occur after the first or second dose of the vaccine [181,182].

Other cutaneous manifestations associated with Moderna, Pfizer, and AstraZeneca vaccines include urticaria, morbilliform rash, papulovesicular rash, pityriasis rosea-like rash, and purpuric reactions [37]. (Figure 4C) Furthermore, eruptions of rosacea have been documented after receiving COVID-19 vaccinations. Figure 4D,E illustrates a documented case of rosacea in a 45-year-old woman after receiving the Sinovac-CoronaVac COVID-19 DNA vaccine. Other reported cases of morbilliform rash progressed to severe dermatological reactions, including erythroderma, bullous pemphigoid, acute generalized exanthematous pustulosis, vasculitis, and urticaria [37]. The patterns of vaccine-induced rashes are similar to rashes described in association with SARS-CoV-2 infection [38,48,183]. It has been proposed that the host immune response, rather than direct SARS-CoV-2 damage, can cause these skin lesions [37,161]. Additionally, DTHR to vaccine excipients has been shown to play a role in the pathogenesis of these skin lesions [37].

## 7. Stress-Induced Dermatological Conditions

The COVID-19 pandemic has been a major disturbance to people’s lives globally. With lockdowns and restricted quarantine measures, people worldwide are concerned about their safety, job security, lack of access to treatment, commodities, and adverse socioeconomic consequences [186]. An increased prevalence of psychiatric illnesses during the COVID-19 pandemic has been reported, and follows a similar pattern observed in the populations following natural disasters [186]. Pandemic-associated psychiatric conditions include anxiety disorders, depression, post-traumatic stress disorder (PTSD) symptoms, sleep disorders, somatic symptoms, and suicidal behavior [186,187,188,189]. Furthermore, epidemiological data conducted during previous pandemics (e.g., severe acute respiratory syndrome (SARS), Ebola, H1N1 influenza, Middle East Respiratory Syndrome (MERS), equine influenza) suggest an increased incidence of psychopathological disorders. Psychological stress, anxiety, and depression have been associated with an increased exacerbation of stress-responsive dermatological conditions [190]. As such, it can be logically presumed that a higher incidence of comorbid psychopathology and psychodermatological disorders will be observed during the COVID-19 pandemic.

Likewise, it is prudent for dermatologists to thoroughly evaluate patients with a history of psychological illnesses or psychiatric diagnoses. When identified, these patients should be referred for appropriate psychosocial support to improve psychosocial outcomes and reduce potential dermatological exacerbations. The consistent epidemiological trend of psychopathological cutaneous disorders justifies a more specialized standard of care for at-risk individuals. Further research is recommended to help identify potential strategies to improve the identification and management of at-risk individuals.

Psychodermatological disorders are classified into four categories summarized in Table 4 [186,191].

The following psychodermatological conditions can be classified as either psychophysiological dermatoses or dermatoses leading to psychosocial comorbidities.

### 7.1. Telogen Effluvium

**Figure 5:**
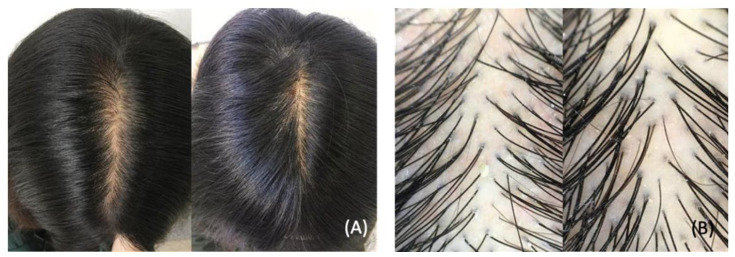
Stress-Related Dermatologic Manifestations during the COVID-19 Pandemic. (**A**,**B**) Telogen effluvium (coined “COVID scalp”) depicting thinning of hair along midline part [192].

Telogen effluvium (TE) is a common, self-limiting hair loss condition usually seen in women with a history of a recent stressor. Stressors associated with TE include systemic disease, infections, stressful events, drugs, nutritional deficiencies, postpartum hormonal changes, and major surgeries [44]. Figure 5 illustrates a clinical presentation of telogen effluvium related to COVID-19 [192]. TE usually presents three months following the onset of the stressor. It is classified as acute (hair loss lasting up to six months) or chronic (hair loss exceeding six months) [193]. Coined as “COVID scalp,” increased cases of TE have been associated with the COVID-19 pandemic [194]. The cause of TE during the COVID-19 pandemic is multifactorial, and it is thought to be stress-induced or a direct manifestation of the inflammatory process during the infection phase.

A study in an ambulatory dermatology clinic in New York City identified women (median age 55) with hair loss that commenced weeks to months post COVID-19 infection. When diagnosed, the identified cohort did not present with any new medical diagnoses. Additionally, these women lacked autoimmune disorders, vitamin deficiencies, or hormonal abnormalities. Physical examinations revealed non-cicatricial loss of hair volume, pronounced thinning, and positive hair-pull tests, consistent with the diagnosis of TE [195].

Another study in Italy identified 25 female patients (age 21 and 54) with a previous diagnosis of TE who presented with exacerbated hair loss symptoms after COVID-19 infection. The worsening of hair loss was associated with the psychosocial stress related to severe lockdown conditions [44]. Turkmen et al. conducted a web-based questionnaire about pre- and post-pandemic TE, alopecia areata, and seborrheic dermatitis. TE was recorded in 27.9% of 563 individuals, possibly due to psychological distress [43].

Increased psychosocial tensions and stressors associated with the COVID-19 pandemic have contributed to exacerbation or a new onset of TE in the previous studies. Contrastingly, Rossi et al. proposed that inflammatory response and direct viral damage to hair follicles contribute to the pathogenesis of TE observed in COVID-19 infections. Rossi hypothesized that viral interferons, IL-6 and IL-4, promote inflammation and apoptosis of hair follicles. Additionally, direct viral damage to hair follicles may contribute to the early onset of COVID-19 TE [42]. These studies highlight the impact of the pandemic on the development or worsening of TE. Further investigations are needed to elucidate the underlying pathomechanism between SARS-CoV2 and TE.

### 7.2. Psoriasis

Psoriasis is a relatively common chronic inflammatory condition of the skin affecting individuals with an underlying genetic predisposition [196]. Multiple triggers associated with psoriasis include infections, mechanical irritation, and drugs [197,198,199]. Guttate psoriasis has been documented as a rare dermatological sequela of COVID-19 infection. Documented infection-related guttate psoriasis is presented in patients with pre-existing psoriasis [200]. Exacerbation of psoriasis can be triggered by stress and anxiety. Mahil et al. conducted a global cross-sectional study from 86 countries to identify factors associated with worsening psoriasis during the COVID-19 pandemic. The study included 4043 individuals with a pre-existing diagnosis of psoriasis. Approximately 43% of these individuals reported psoriasis flares during the pandemic. Factors that have been associated with worsened psoriasis include poor mental health, female gender, obesity, and shielding. Also, they found specific targeted and systemic therapies (e.g., TNF-α inhibitors, IL-17 inhibitors, IL-23 inhibitors, and apremilast) are inversely associated with aggravation of psoriasis [45]. This survey underscores the impact of pandemic-related psychological stress and its impact on the worsening of psoriasis.

Kuang et al. conducted a web-based survey in China to assess loss of income and restriction of outdoor activity during the COVID-19 pandemic and its consequential effect on psoriasis exacerbation. They found that 43.7% (*n* = 405) of 926 questionnaires collected reported an aggravation of psoriasis. Outdoor activity restriction and loss of income were positively associated with the worsening of psoriasis. Researchers theorize that psychological distress due to a loss of income and outdoor activity restrictions have contributed to psoriasis flares in these patients [201].

### 7.3. Eczema, Urticaria, and Atopic dermatitis

Increased psychosocial stress during the COVID-19 pandemic has been shown to aggravate stress-responsive skin conditions such as eczema, urticaria, and atopic dermatitis [202]. Limited data exists regarding these dermatological conditions and its relationship with pandemic-induced stress. A worsening of these skin conditions can be secondary to excessive hand hygiene practices during the pandemic [203,204]. Individuals who endure high-stress levels during the pandemic, such as healthcare providers, resort to vigorous hand hygiene practices. Therefore, more research is needed to establish an association between pandemic-related stresses versus excessive hand hygiene in the exacerbation of the aforementioned skin conditions.

## 8. Role of Dermatologists in the COVID-19 Pandemic

### 8.1. Teledermatology

Amid the pandemic, dermatologists have played a crucial role in providing patients with timely and effective dermatologic care. However, the COVID-19 pandemic has led to significant adaptations, such as teledermatology. Teledermatology was utilized before the COVID-19 pandemic, albeit infrequently. This modality existed primarily as a store-and-forward model with photos viewed at a future time [205]. During the COVID-19 era, teledermatology evolved to provide patients with better access to dermatological care remotely, thereby reducing SARS-CoV-2 infection risk. The need for delivery of safe care, policy changes in insurance reimbursement for telemedicine visits, medicolegal liabilities, and reduced licensing restrictions have allowed for the rapid expansion of teledermatology and live-interactive synchronous video visits [206].

Through video teleconferencing, dermatologists can provide remote consultations, diagnosing visible skin conditions, and providing appropriate treatments [207]. Teledermatology removes the burden of traveling to the physician’s office, especially for elderly patients with limited mobility and patients with limited access to transportation. Additionally, teledermatology is equally effective as conventional in-person dermatology visits, with diagnostic accuracy in the 70th percentile [208,209,210]. Therefore, teledermatology is best suited for use in straightforward cases of common dermatologic diseases such as acne, rosacea, psoriasis, and eczema. In addition to its clinical effectiveness, teledermatology encounters are cost-effective and allow for efficient case triage [211]. This pragmatic modality will enable dermatologists to conveniently assess acute cases and reduce avoidable visits to urgent care or emergency departments [211,212].

Despite its numerous advantages, teledermatology has its limitations. In-person visits are irreplaceable when performing full-body examinations to screen for potentially cancerous or pre-cancerous lesions [213]. Without full-body examination, such lesions are likely to be missed during a teledermatology encounter [213]. However, multiple studies have demonstrated that teledermatology expedited vital care for patients with suspicious cancerous lesions [214,215,216,217]. A research study conducted at a Veterans Health Administration (VHA) dermatology clinic revealed that teledermatology reduces the time for consultations, biopsies, and excisions [215]. Other potential pitfalls of teledermatology are outlined in Table 5.

Despite its limitations, teledermatology continues to play an integral role in dermatology practice as society evolves through the pandemic.

### 8.2. Clinical Practice Guidelines

New data and information regarding the COVID-19 infection are in constant development. Consequently, dermatologists have re-evaluated clinical practice guidelines more frequently to remain consistent with evolving recommendations. Specifically, there has been controversy regarding guidelines for the use of immunomodulators and immunosuppressants in COVID-19 patients with autoimmune and inflammatory conditions such as atopic dermatitis, psoriasis, and pemphigus vulgaris. Initially, practitioners expressed concern that such medications could increase the risk of contracting COVID-19 [218,219]. However, the American Academy of Dermatology (AAD) and the American College of Rheumatology (ACR) have recommended the continuation of treatment for most immunomodulatory medications. Concerns specifically regarding prednisone and JAK-inhibitors in the setting of COVID-19 have caused dermatologists to reassess their treatment protocols and consider other treatment alternatives. Dermatologists continue to weigh the risks and benefits of continuation or initiation of immunosuppressants based on factors such as age, comorbidities, genetic history, and severity of skin disease [220].

## 9. Discussion

The COVID-19 pandemic has caused an insurmountable disturbance on a global scale. Several heterogeneous factors impact the severity and mortality of Coronavirus Disease. Comorbidities such as hypertension, diabetes mellitus, and cardiovascular diseases have been associated with severe cases of COVID-19 infection [221]. In addition to respiratory symptoms, numerous cases of cutaneous manifestations related to SARS-CoV-2 infection have been reported.

In efforts to classify these lesions, the AAD’s COVID-19 Dermatology Registry offers a morphological classification of COVID-19-related cutaneous manifestations. However, some cutaneous lesions were diagnosed and labeled by non-dermatologists. Moreover, greater than half of the new-onset dermatological conditions in the setting of COVID-19 were reported by non-dermatologists, such as non-dermatologic physicians, nurses, and physician assistants [23]. Consequently, there is a potential for morphologic misclassification and inconsistent description of cutaneous findings. Additional AAD registry limitations involve selective reporting and a lack of consistent measures [21].

To address confusion regarding the classification of cutaneous manifestations related to COVID-19, Suchonwanit et al. proposed the classification of skin disease into two main groups based on mechanistic patterns [222]. The first group includes viral exanthems triggered by an immune response to viral nucleotides, such as morbilliform rash and urticaria. The second group organizes cutaneous eruptions secondary to systemic manifestations due to COVID-19 infection, such as vasculitis and thrombotic vasculopathy. Though relatively broad, this dichotomy is a helpful first step in the dermatological classification of COVID-19-related cutaneous conditions and associated treatments [222].

Skin eruptions in COVID-19 patients have been labeled and categorized based on morphological similarities to their viral counterparts, such as “varicella-like” or “morbilliform-like” exanthems [223]. Other skin lesions in COVID-19 patients present similarly to skin eruptions seen in vasculitides such as papulosquamous eruptions, retiform purpura, livedo reticularis, and more [223]. Many of the discussed dermatological morphologies present with other viral infections. Therefore, the dermatological morphologies may not provide specific insight into COVID-specific pathophysiology or individualized treatment targets. As such, it is challenging to establish COVID-19 as the definitive cause of these skin lesions. Further research is needed to establish a definitive etiology.

Authors have proposed subtle clinical clues to differentiate COVID-related rashes from other viral or idiopathic rashes. For example, one case series reported palatal petechiae or macules in six out of twenty-one patients with known a COVID-19 diagnosis and cutaneous rash. These exanthems suggested viral etiology, yet provided no specificity to COVID-19 infection [223]. Potential etiologies for COVID-19-related cutaneous manifestations includes hypersensitivity of the immune system in response to the SARS-CoV-2 RNA, cytokine release, microthrombi formation, and vasculitis development. Limited understanding of the mechanism behind these dermatological manifestations remains an obstacle. However, the specific etiologies of cutaneous manifestations related to SARS-CoV-2 continue to be analyzed.

Multiple established associations in the scientific literature offer valuable insights into plausible mechanisms behind cutaneous manifestations of SARS-CoV-2. One proposed pathomechanism explains the presence of ACE2 in keratinocytes [19]. Another promising association describes the significance of androgen levels in the increased expression of the TMPRSS2 gene [13,14].

ACE2 receptors are found in multiple tissues throughout the body including the skin. In conjunction, the presence of ACE2 receptors in keratinocytes and high androgen levels have been shown to play a significant role in the development of skin lesions [13,14,19]. As such, high levels of androgens combined with ACE2 distribution in the skin can provide invaluable clinical insight into the pathomechanism of skin manifestations.

Androgens have been shown to uniquely activate the TMPRSS2 gene, which promotes ACE2 receptors for the binding of the spike protein and subsequent entry of the virus into host cells. A strong association between increased androgens and increased TMPRSS2 gene expression has been identified in the literature [13,14]. This correlation helps explain the greater susceptibility to COVID-19 in males, as males typically maintain higher levels of androgens. This association helps clarify the less symptomatic disease in children with overall low expressions of androgen receptors [7,13,14,15,16,17].

Additional susceptibility to COVID-19 has been strongly correlated with low vitamin D levels. Studies continue to identify and analyze the increased severity of COVID-19 infection with concomitant low vitamin D levels [224,225,226]. Vitamin D can be administered in various forms including (but not limited to) cholecalciferol, calcifediol, and calcitriol. Vitamin D supplementation has been proposed to reduce the risk of COVID-19 infection through multiple protective mechanisms. Such mechanisms include modulation of the host immune system, upregulation of ACE2 concentration, vitamin D receptor activation [226], reduction in endothelial damage, and reduction in proinflammatory cytokines [224,225,226].

Research studies have demonstrated that vitamin D receptors (VDRs) are expressed in high concentration levels in cuboidal alveolar type II cells (ACII) within the pulmonary system. Calcitriol, also known as 1,25 dihydroxyvitamin D (1,25(OH)_2_ D_3_), binds to VDRs in ACII cells. Calcitriol binding activates multiple intracellular signals which inhibit inflammatory cytokines and chemokines involved in Acute Respiratory Distress Syndrome (ARDS) [226].

Additional investigations have revealed that vitamin D signaling pathways prevent pulmonary vessel constriction, a manifestation associated with increased COVID-19 mortality [224,225,226]. VDR activation promotes vasodilatory effects through two mechanisms: inhibitions of angiotensin II (a potent vasoconstrictor) and upregulation of ACE2. Decreased expression of angiotensin II promotes pulmonary vasodilation [224,225,226]. Additionally, the induction of ACE2 expression in pulmonary tissues further dampens the effect of angiotensin II, thereby reducing respiratory distress symptoms. Prevention of pulmonary vasoconstriction greatly improves respiratory symptoms associated with SARS-CoV-2 infection. Therefore, ACE2 acts as an anti-inflammatory factor in the etiology of ARDS [224,225,226]. VDR activation has also been shown to inhibit Skp2 protein, which is utilized by SARS-CoV-2 to replicate inside cells. Thus, the binding of calcitriol and VDR activation reduce viral replication in pulmonary tissues and reduce the disease severity of COVID-19 [226].

Calcifediol, a vitamin D3 analog, rapidly increases serum levels of vitamin D 25-hydroxyvitamin D (25-OH-D), thereby promoting the protective properties associated with vitamin D [225]. A parallel randomized open label, double-masked clinical trial evaluated the effect of calcifediol on the severity of COVID-19 disease. The randomized clinical trial was conducted on 76 consecutive patients hospitalized with COVID-19 infection. All 76 patients clinically presented with acute respiratory infections, confirmed by radiographic patterns of viral pneumonia. Likewise, all 76 patients tested positive for SARS-CoV-2 through PCR tests. Lastly, all 76 patients were confirmed for appropriate hospital admission via the CURB65 Severity Scale [225]. All hospitalized patients received the best available therapy at the same standard of care. Of the 50 patients treated with calcifediol, one patient required intensive care unit (ICU) admission, and of the 26 untreated patients, 13 patients required ICU admission, with two deaths in the ICU. The remaining 11 untreated patients who did not receive calcifediol were discharged. Of all 50 patients treated with calcifediol, none died, and all patients were discharged with no complications [225].

A larger-scale observational cohort study with 930 patients also revealed significantly reduced ICU admissions and mortality rates associated with early vitamin D administration [227]. These clinical trials underscore the clinical value of vitamin D in a significant reduction in disease severity and disease mortality [225,227].

With regards to infection-related cutaneous manifestations, most conditions have been attributed to the host’s inflammatory response to SARS-CoV-2. Likewise, the immune-boosting, anti-inflammatory properties of vitamin D alleviate and reduce the spread of cutaneous lesions [224,225,226,227]. In addition, vitamin D bioavailability and efficacy have been shown to increase with magnesium supplementation. Magnesium has been shown to facilitate vitamin D-related processes by activating vitamin D processing enzymes [228].

Vitamin D and magnesium supplementation are cost-effective measures that help prevent infection, reduce disease severity, and improve prognosis [228]. Likewise, patient education and adherence are essential factors for favorable clinical outcomes. Therefore, further investigations are warranted to identify the ideal maintenance dosage of vitamin D and magnesium to prevent infection. Additional research is recommended to elucidate an effective dosing regimen to reduce infection-related cutaneous lesions.

Throughout the pandemic, clinical management for most COVID-19-associated and non-COVID-19 cutaneous manifestations have been similar in nature. For example, chilblain-like lesions in COVID-19 patients do not require treatment, but topical corticosteroids can relieve discomfort [62,64,229]. Other rashes, such as varicelliform-like/vesicular lesions, are self-limiting and therefore do not require treatment [48,102]. In contrast, maculopapular and urticarial eruptions can occur concomitantly or separately in moderate to severe cases of COVID-19. These lesions present with pruritus and pain which necessitates prompt treatment with therapeutic agents such as topical corticosteroids, oral antihistamines, oral corticosteroids, and vitamin C [48,230,231,232,233]. In addition to rash therapies, early treatment interventions have been shown to improve the overall prognosis for infected patients and reduce patient mortality [234,235].

Severe COVID-19 infections have been shown to induce systemic vasculopathies and hypercoagulable states. These vasculopathies and hypercoagulable states are associated with cutaneous eruptions, such as purpuric, petechial, or livedoid lesions [236]. Addressing hematologic disorders is essential in the management and treatment of these lesions. Treatment recommendations include supportive measures and anticoagulant therapy for systemic disease [237,238,239]. The remainder of treatments for most COVID-19-related dermatologic manifestations are aimed at symptom control with topical management or oral antihistamines. Likewise, watchful waiting for anticipated self-resolution plays a pragmatic role in dermatological treatments.

The COVID-19 pandemic and the strict quarantine measures have caused significant psychosocial distress. The pandemic has disrupted people’s daily lives and routines. Resultantly, many individuals have developed new or worsening pre-existing dermatological conditions, such as telogen effluvium, psoriasis, eczema, urticaria, and atopic dermatitis. Exacerbated lesions require supportive management, targeted treatment for underlying issues, and relevant psychosocial support. Additionally, interdisciplinary treatment involving mental health professionals should be implemented to help relieve stress, anxiety, and depression.

As the pandemic continues, strict measures and mandates have been implemented to promote mass vaccination. With increased immunizations, additional cases of anaphylaxis and other allergic reactions such as COVID toes, COVID arm, and urticaria have been documented in response to immunizations [37,38,175,176,177,180]. It has been proposed that lipid nanoparticles (LNPs) containing messenger RNA vaccines trigger allergic and anaphylactic reactions [240]. LNPs are composed of positively charged lipids at low pH to stabilize the messenger RNA [241]. Likewise, LNPs contain high amounts of polyethylene glycol (PEG), a highly hydrophilic molecule [239,242]. PEG helps to increase the hydrophilicity of LNPs and stabilize the mRNA. However, PEG within LNPs has been shown to trigger inflammatory responses through complement-mediated and direct mast cell activation [240].

Furthermore, when inoculated into the bloodstream, LNPs trigger nonclassical allergic reactions in certain patients. Such reactions involve preformed antibodies to PEG and other components of LNPs [240]. Moreover, LNPs destabilize during the freeze and thaw cycle of immunization preparation [240]. When injected, destabilized LNPs release the naked mRNA into the bloodstream. Naked mRNA is proinflammatory and has been shown to induce allergic and anaphylactic reactions [240,242]. Additionally, classical allergic reactions to PEG may be IgE-mediated [240]. As such, skin prick testing with PEG should be performed before receiving the vaccine to avoid anaphylactic reactions [168]. At this time, allergy testing has not been implemented to screen susceptible individuals and should be considered to maximize safety and minimize adverse events.

As evidenced in this review, the COVID-19 pandemic has triggered a significant range of dermatologic sequela. Several years into the pandemic, there is still much to learn and understand. As more information is collected and assessed, our comprehension of the pathogenesis and treatment of dermatologic manifestations will continue to evolve and guide the dermatological standard of care.

## 10. Conclusions

The COVID-19 pandemic has posed considerable challenges across the entirety of medicine. Various factors surrounding the pandemic have resulted in both novel and a multitude of other dermatological manifestations. Such factors include COVID-19 infection, COVID-19 vaccination, personal protective equipment, and psychological distress related to the pandemic.

A multidisciplinary approach is ideal when managing COVID-19 patients with dermatological conditions. Psychosocial factors include impacts of quarantine, restricted measures, and major life changes. Providers such as psychiatrists and therapists are needed to address the root cause of stress-induced cutaneous manifestations. In more severe cases, coordinated care with pulmonologists, intensivists, and cardiologists can provide a comprehensive treatment plan to resolve infections and mitigate risk factors.

Cutaneous manifestations from the COVID-19 pandemic continue to increase. These dermatological conditions warrant specialized care and detailed evaluations by board-certified dermatologists. Dermatologists continue to play a vital role in the clinical guidelines and classification of pandemic-related lesions. Given the evolutionary nature of the pandemic, intentional observation and research are prudent in the diagnosis, management, and treatment of these cutaneous manifestations.

The COVID-19 pandemic has disrupted society in a multifaceted way and likewise warrants a heterogeneous approach to restore public health. Cost-effective prevention is prudent on a global scale. Discussions on established vitamin supplementation protocols should be integrated into patient education and the overall standard of care in all disciplines. Early treatment regimens and timely prophylaxis have been shown to improve prognosis and reduce further infection-related sequelae. Novel measures on vaccine risk reduction should improve outcomes and maximize safety. Robust investigations are necessary to identify underlying dermatological pathomechanisms and improve lesion diagnosis. Data collection will help reveal pertinent risk factors and boost public health outcomes. Such studies will reduce disease burden and optimize quality of life as society continues to adapt and adjust to life after SARS-CoV-2.

## Figures and Tables

**Table 4:** Classification of Psychodermatological Disorders.

	Definition	Examples
Psychophysiological dermatoses	Group of skin diseases that are exacerbated by psychological stress	–Chronic urticaria –Telogen effluvium –Alopecia areata –Psoriasis –Eczema
Primary cutaneous psychopathology	Group of skin symptoms of skin-inflicted skin lesion as a result of an underlying psychiatric illness without primary dermatosis	–Delusional infestations –Factitious disorders –Skin picking
Cutaneous sensory disorders	Group of heterogeneous clinical situations that present with disagreeable skin sensations, pain, or negative sensory symptoms	–Hypoesthesia –Numbness –Allodynia –Stinging –Burning –Itching
Dermatoses leading to psychosocial comorbidities	Disfiguring skin conditions that contribute to psychosocial distress	–Telogen effluvium –Alopecia areata –Psoriasis

**Table 5:** Obstacles in Teledermatology.

Obstacles	Examples
Difficulty in Diagnosis	Inadequate clinical history, lack of context Inability to palpate lesions Inability to perform complete skin exams Poor image quality Technical difficulties Incorrect clinical photographs Lack of imaging or technique standards Lack of access to prior medication records
Compromised Continuity of Care	No continuity and/or longitudinal care Inability to consult patient in person Obstacles in obtaining diagnostic or lab tests Lack of communication with primary care providers Less integration into hospital health systems
Policy/Legal Risk	Medico-legal/malpractice risk Security breaches HIPAA * violations **
Services/Costs Reimbursements	Lack of universal payments ** Limited reimbursements: Medicare/Medicaid ** Lack of universal private payer parity Maintenance costs for individual systems

* Health Insurance Portability and Accountability Act of 1996. ** USA jurisdiction.

## Data Availability

Not applicable.

## References

[1] Zhu N., Zhang D., Wang W., Li X., Yang B., Song J., Zhao X., Huang B., Shi W., Lu R. (2020). A Novel Coronavirus from Patients with Pneumonia in China, 2019. N. Engl. J. Med..

[2] Cucinotta D., Vanelli M. (2020). WHO Declares COVID-19 a Pandemic. Acta Biomed..

[3] Worldmeter Coronavirus Cases. https://www.worldometers.info/coronavirus/.

[4] Li J., Jia H., Tian M., Wu N., Yang X., Qi J., Ren W., Li F., Bian H. (2022). SARS-CoV-2 and Emerging Variants: Unmasking Structure, Function, Infection, and Immune Escape Mechanisms. Front. Cell Infect. Microbiol..

[5] Liu J., Li Y., Liu Q., Yao Q., Wang X., Zhang H., Chen R., Ren L., Min J., Deng F. (2021). SARS-CoV-2 cell tropism and multiorgan infection. Cell Discov..

[6] Conceicao C., Thakur N., Human S., Kelly J.T., Logan L., Bialy D., Bhat S., Stevenson-Leggett P., Zagrajek A.K., Hollinghurst P. (2020). The SARS-CoV-2 Spike protein has a broad tropism for mammalian ACE2 proteins. PLoS Biol..

[7] Zou X., Chen K., Zou J., Han P., Hao J., Han Z. (2020). Single-cell RNA-seq data analysis on the receptor ACE2 expression reveals the potential risk of different human organs vulnerable to 2019-nCoV infection. Front. Med..

[8] Qi F., Qian S., Zhang S., Zhang Z. (2020). Single cell RNA sequencing of 13 human tissues identify cell types and receptors of human coronaviruses. Biochem. Biophys. Res. Commun..

[9] Zhang H., Kang Z., Gong H., Xu D., Wang J., Li Z., Li Z., Cui X., Xiao J., Zhan J. (2020). Digestive system is a potential route of COVID-19: An analysis of single-cell coexpression pattern of key proteins in viral entry process. Gut.

[10] Zhao Y., Zhao Z., Wang Y., Zhou Y., Ma Y., Zuo W. (2020). Single-Cell RNA Expression Profiling of ACE2, the Receptor of SARS-CoV-2. Am. J. Respir. Crit. Care Med..

[11] Kabbani N., Olds J.L. (2020). Does COVID19 Infect the Brain? If So, Smokers Might Be at a Higher Risk. Mol. Pharmacol..

[12] Xu H., Zhong L., Deng J., Peng J., Dan H., Zeng X., Li T., Chen Q. (2020). High expression of ACE2 receptor of 2019-nCoV on the epithelial cells of oral mucosa. Int. J. Oral. Sci..

[13] Kaya G., Kaya A., Saurat J.H. (2020). Clinical and Histopathological Features and Potential Pathological Mechanisms of Skin Lesions in COVID-19: Review of the Literature. Dermatopathology.

[14] Mjaess G., Karam A., Aoun F., Albisinni S., Roumeguère T. (2020). COVID-19 and the male susceptibility: The role of ACE2, TMPRSS2 and the androgen receptor. Prog. Urol..

[15] Huang C., Wang Y., Li X., Ren L., Zhao J., Hu Y., Zhang L., Fan G., Xu J., Gu X. (2020). Clinical features of patients infected with 2019 novel coronavirus in Wuhan, China. Lancet.

[16] Richardson S., Hirsch J.S., Narasimhan M., Crawford J.M., McGinn T., Davidson K.W., Barnaby D.P., Becker L.B., Chelico J.D., The Northwell COVID-19 Research Consortium (2020). Presenting Characteristics, Comorbidities, and Outcomes among 5700 Patients Hospitalized with COVID-19 in the New York City Area. JAMA.

[17] Wang D., Hu B., Hu C., Zhu F., Liu X., Zhang J., Wang B., Xiang H., Cheng Z., Xiong Y. (2020). Clinical Characteristics of 138 Hospitalized Patients with 2019 Novel Coronavirus-Infected Pneumonia in Wuhan, China. JAMA.

[18] Gupta A., Madhavan M.V., Sehgal K., Nair N., Mahajan S., Sehrawat T.S., Bikdeli B., Ahluwalia N., Ausiello J.C., Wan E.Y. (2020). Extrapulmonary manifestations of COVID-19. Nat. Med..

[19] Xue X., Mi Z., Wang Z., Pang Z., Liu H., Zhang F. (2021). High Expression of ACE2 on Keratinocytes Reveals Skin as a Potential Target for SARS-CoV-2. J. Investig. Dermatol..

[20] Wei C., Friedman A.J. (2020). COVID-19 Pandemic: Are There Unique Cutaneous Manifestations in Patients Infected With SARS-CoV-2?. J. Drugs Dermatol..

[21] Freeman E.E., McMahon D.E., Fitzgerald M.E., Fox L.P., Rosenbach M., Takeshita J., French L.E., Thiers B.H., Hruza G.J. (2020). The American Academy of Dermatology COVID-19 registry: Crowdsourcing dermatology in the age of COVID-19. J. Am. Acad. Dermatol..

[22] Freeman E.E., Chamberlin G.C., McMahon D.E., Hruza G.J., Wall D., Meah N., Sinclair R., Balogh E.A., Feldman S.R., Lowes M.A. (2021). Dermatology COVID-19 Registries: Updates and Future Directions. Dermatol. Clin..

[23] Freeman E.E., McMahon D.E., Lipoff J.B., Rosenbach M., Kovarik C., Desai S.R., Harp J., Takeshita J., French L.E., Lim H.W. (2020). The spectrum of COVID-19-associated dermatologic manifestations: An international registry of 716 patients from 31 countries. J. Am. Acad. Dermatol..

[24] Marzano A.V., Genovese G., Moltrasio C., Gaspari V., Vezzoli P., Maione V., Misciali C., Sena P., Patrizi A., Offidani A. (2021). The clinical spectrum of COVID-19-associated cutaneous manifestations: An Italian multicenter study of 200 adult patients. J. Am. Acad. Dermatol..

[25] Lavery M.J., Bouvier C.A., Thompson B. (2021). Cutaneous manifestations of COVID-19 in children (and adults): A virus that does not discriminate. Clin. Dermatol..

[26] Young T.K., Shaw K.S., Shah J.K., Noor A., Alperin R.A., Ratner A.J., Orlow S.J., Betensky R.A., Shust G.F., Kahn P.J. (2021). Mucocutaneous Manifestations of Multisystem Inflammatory Syndrome in Children During the COVID-19 Pandemic. JAMA Dermatol..

[27] Naka F., Melnick L., Gorelik M., Morel K.D. (2021). A dermatologic perspective on multisystem inflammatory syndrome in children. Clin. Dermatol..

[28] Damiani G., Gironi L.C., Grada A., Kridin K., Finelli R., Buja A., Bragazzi N.L., Pigatto P.D.M., Savoia P. (2021). COVID-19 related masks increase severity of both acne (maskne) and rosacea (mask rosacea): Multi-center, real-life, telemedical, and observational prospective study. Dermatol. Ther..

[29] Rudd E., Walsh S. (2021). Mask related acne (“maskne”) and other facial dermatoses. BMJ.

[30] Yu J., Chen J.K., Mowad C.M., Reeder M., Hylwa S., Chisolm S., Dunnick C.A., Goldminz A.M., Jacob S.E., Wu P.A. (2021). Occupational dermatitis to facial personal protective equipment in health care workers: A systematic review. J. Am. Acad. Dermatol..

[31] Lin P., Zhu S., Huang Y., Li L., Tao J., Lei T., Song J., Liu D., Chen L., Shi Y. (2020). Adverse skin reactions among healthcare workers during the coronavirus disease 2019 outbreak: A survey in Wuhan and its surrounding regions. Br. J. Dermatol..

[32] Lan J., Song Z., Miao X., Li H., Li Y., Dong L., Yang J., An X., Zhang Y., Yang L. (2020). Skin damage among health care workers managing coronavirus disease-2019. J. Am. Acad. Dermatol..

[33] Zhou N.Y., Yang L., Dong L.Y., Li Y., An X.J., Yang J., Yang L., Huang C.Z., Tao J. (2020). Prevention and Treatment of Skin Damage Caused by Personal Protective Equipment: Experience of the First-Line Clinicians Treating 2019-nCoV Infection. Int. J. Dermatol. Venereol..

[34] Vasireddy D., Atluri P., Malayala S.V., Vanaparthy R., Mohan G. (2021). Review of COVID-19 Vaccines Approved in the United States of America for Emergency Use. J. Clin. Med. Res..

[35] Centers for Disease Control and Prevention Interim Clinical Considerations for Use of COVID-19 Vaccines. https://www.cdc.gov/vaccines/covid-19/clinical-considerations/covid-19-vaccines-us.html#Appendix-C.

[36] Meara A.S., Silkowski M., Quin K., Jarjour W. (2021). A Case of Chilblains-like Lesions Post SARS-CoV-2 Vaccine?. J. Rheumatol..

[37] Català A., Muñoz-Santos C., Galván-Casas C., Roncero Riesco M., Revilla Nebreda D., Solá-Truyols A., Giavedoni P., Llamas-Velasco M., González-Cruz C., Cubiró X. (2022). Cutaneous reactions after SARS-CoV-2 vaccination: A cross-sectional Spanish nationwide study of 405 cases. Br. J. Dermatol..

[38] McMahon D.E., Amerson E., Rosenbach M., Lipoff J.B., Moustafa D., Tyagi A., Desai S.R., French L.E., Lim H.W., Thiers B.H. (2021). Cutaneous reactions reported after Moderna and Pfizer COVID-19 vaccination: A registry-based study of 414 cases. J. Am. Acad. Dermatol..

[39] Glowacz F., Schmits E. (2020). Psychological distress during the COVID-19 lockdown: The young adults most at risk. Psychiatry Res..

[40] Wang Y., Kala M.P., Jafar T.H. (2020). Factors associated with psychological distress during the coronavirus disease 2019 (COVID-19) pandemic on the predominantly general population: A systematic review and meta-analysis. PLoS ONE.

[41] Kim H.H., Jung J.H. (2021). Social Isolation and Psychological Distress during the COVID-19 Pandemic: A Cross-National Analysis. Gerontologist.

[42] Rossi A., Magri F., Sernicola A., Michelini S., Caro G., Muscianese M., Di Fraia M., Chello C., Fortuna M.C., Grieco T. (2021). Telogen Effluvium after SARS-CoV-2 Infection: A Series of Cases and Possible Pathogenetic Mechanisms. Skin Appendage Disord..

[43] Turkmen D., Altunisik N., Sener S., Colak C. (2020). Evaluation of the effects of COVID-19 pandemic on hair diseases through a web-based questionnaire. Dermatol. Ther..

[44] Rivetti N., Barruscotti S. (2020). Management of telogen effluvium during the COVID-19 emergency: Psychological implications. Dermatol. Ther..

[45] Mahil S.K., Yates M., Yiu Z., Langan S.M., Tsakok T., Dand N., Mason K.J., McAteer H., Meynell F., Coker B. (2021). Describing the burden of the COVID-19 pandemic in people with psoriasis: Findings from a global cross-sectional study. J. Eur. Acad. Dermatol. Venereol..

[46] Yazdany J., Manno R.L., Papadakis M.A., McPhee S.J., Rabow M.W., McQuaid K.R. (2022). Delayed Hypersensitivity. Current Medical Diagnosis & Treatment 2022.

[47] Giavedoni P., Podlipnik S., Pericàs J.M., Fuertes de Vega I., García-Herrera A., Alós L., Carrera C., Andreu-Febrer C., Sanz-Beltran J., Riquelme-Mc Loughlin C. (2020). Skin Manifestations in COVID-19: Prevalence and Relationship with Disease Severity. J. Clin. Med..

[48] Galván Casas C., Català A., Carretero Hernández G., Rodríguez-Jiménez P., Fernández-Nieto D., Rodríguez-Villa Lario A., Navarro Fernández I., Ruiz-Villaverde R., Falkenhain-López D., Llamas Velasco M. (2020). Classification of the cutaneous manifestations of COVID-19: A rapid prospective nationwide consensus study in Spain with 375 cases. Br. J. Dermatol..

[49] Do M.H., Stewart C.R., Harp J. (2021). Cutaneous Manifestations of COVID-19 in the Inpatient Setting. Dermatol. Clin..

[50] Rekhtman S., Tannenbaum R., Strunk A., Birabaharan M., Wright S., Grbic N., Joseph A., Lin S.K., Zhang A.C., Lee E.C. (2021). Eruptions and related clinical course among 296 hospitalized adults with confirmed COVID-19. J. Am. Acad. Dermatol..

[51] Askin O., Altunkalem R.N., Altinisik D.D., Uzuncakmak T.K., Tursen U., Kutlubay Z. (2020). Cutaneous manifestations in hospitalized patients diagnosed as COVID-19. Dermatol. Ther..

[52] Català A., Galván-Casas C., Carretero-Hernández G., Rodríguez-Jiménez P., Fernández-Nieto D., Rodríguez-Villa A., Navarro-Fernández Í., Ruiz-Villaverde R., Falkenhain-López D., Llamas-Velasco M. (2020). Maculopapular eruptions associated to COVID-19: A subanalysis of the COVID-Piel study. Dermatol. Ther..

[53] Ghimire K., Adhikari N. (2021). Morbilliform rashes in a patient with COVID-19 infection: A case report. JNMA J. Nepal Med. Assoc..

[54] Kulkarni R.B., Lederman Y., Afiari A., Savage J.A., Jacob J. (2020). Morbilliform Rash: An Uncommon Herald of SARS-CoV-2. Cureus.

[55] Fattori A., Cribier B., Chenard M.P., Mitcov M., Mayeur S., Weingertner N. (2021). Cutaneous manifestations in patients with coronavirus disease 2019: Clinical and histological findings. Hum. Pathol..

[56] Ahouach B., Harent S., Ullmer A., Martres P., Bégon E., Blum L., Tess O., Bachmeyer C. (2020). Cutaneous lesions in a patient with COVID-19: Are they related?. Br. J. Dermatol..

[57] Najarian D.J. (2020). Morbilliform exanthem associated with COVID-19. JAAD Case Rep..

[58] Hedrich C.M., Fiebig B., Hauck F.H., Sallmann S., Hahn G., Pfeiffer C., Heubner G., Lee-Kirsch M.A., Gahr M. (2008). Chilblain lupus erythematosus—A review of literature. Clin. Rheumatol..

[59] Su W.P., Perniciaro C., Rogers R.S., White J.W. (1994). Chilblain lupus erythematosus (lupus pernio): Clinical review of the Mayo Clinic experience and proposal of diagnostic criteria. Cutis.

[60] Cappel J.A., Wetter D.A. (2014). Clinical characteristics, etiologic associations, laboratory findings, treatment, and proposal of diagnostic criteria of pernio (chilblains) in a series of 104 patients at Mayo Clinic, 2000 to 2011. Mayo Clin. Proc..

[61] de Masson A., Bouaziz J.D., Sulimovic L., Cassius C., Jachiet M., Ionescu M.A., Rybojad M., Bagot M., Duong T.A., SNDV (French National Union of Dermatologists-Venereologists) (2020). Chilblains is a common cutaneous finding during the COVID-19 pandemic: A retrospective nationwide study from France. J. Am. Acad. Dermatol..

[62] Piccolo V., Neri I., Filippeschi C., Oranges T., Argenziano G., Battarra V.C., Berti S., Manunza F., Fortina A.B., Di Lernia V. (2020). Chilblain-like lesions during COVID-19 epidemic: A preliminary study on 63 patients. J. Eur. Acad. Dermatol. Venereol..

[63] Kolivras A., Dehavay F., Delplace D., Feoli F., Meiers I., Milone L., Olemans C., Sass U., Theunis A., Thompson C.T. (2020). Coronavirus (COVID-19) infection-induced chilblains: A case report with histopathologic findings. JAAD Case Rep..

[64] El Hachem M., Diociaiuti A., Concato C., Carsetti R., Carnevale C., Ciofi Degli Atti M., Giovannelli L., Latella E., Porzio O., Rossi S. (2020). A clinical, histopathological and laboratory study of 19 consecutive Italian paediatric patients with chilblain-like lesions: Lights and shadows on the relationship with COVID-19 infection. J. Eur. Acad. Dermatol. Venereol..

[65] Kanitakis J., Lesort C., Danset M., Jullien D. (2020). Chilblain-like acral lesions during the COVID-19 pandemic (“COVID toes”): Histologic, immunofluorescence, and immunohistochemical study of 17 cases. J. Am. Acad. Dermatol..

[66] Colmenero I., Santonja C., Alonso-Riaño M., Noguera-Morel L., Hernández-Martín A., Andina D., Wiesner T., Rodríguez-Peralto J.L., Requena L., Torrelo A. (2020). SARS-CoV-2 endothelial infection causes COVID-19 chilblains: Histopathological, immunohistochemical and ultrastructural study of seven paediatric cases. Br. J. Dermatol..

[67] Santonja C., Heras F., Núñez L., Requena L. (2020). COVID-19 chilblain-like lesion: Immunohistochemical demonstration of SARS-CoV-2 spike protein in blood vessel endothelium and sweat gland epithelium in a polymerase chain reaction-negative patient. Br. J. Dermatol..

[68] Gambichler T., Reuther J., Stücker M., Stranzenbach R., Torres-Reyes C., Schlottmann R., Schmidt W.E., Hayajneh R., Sriram A., Becker J.C. (2021). SARS-CoV-2 spike protein is present in both endothelial and eccrine cells of a chilblain-like skin lesion. J. Eur. Acad. Dermatol. Venereol..

[69] Ko C.J., Harigopal M., Damsky W., Gehlhausen J.R., Bosenberg M., Patrignelli R., McNiff J.M. (2020). Perniosis during the COVID-19 pandemic: Negative anti-SARS-CoV-2 immunohistochemistry in six patients and comparison to perniosis before the emergence of SARS-CoV-2. J. Cutan. Pathol..

[70] Baeck M., Hoton D., Marot L., Herman A. (2020). Chilblains and COVID-19: Why SARS-CoV-2 endothelial infection is questioned. Br. J. Dermatol..

[71] Freeman E.E., McMahon D.E., Lipoff J.B., Rosenbach M., Desai S.R., Fassett M., French L.E., Lim H.W., Hruza G.J., Fox L.P. (2021). Cold and COVID: Recurrent pernio during the COVID-19 pandemic. Br. J. Dermatol..

[72] Palamaras I., Kyriakis K. (2005). Calcium antagonists in dermatology: A review of the evidence and research-based studies. Dermatol. Online J..

[73] Whitman P.A., Crane J.S. (2022). Pernio.

[74] Kayiran M.A., Akdeniz N. (2019). Diagnosis and treatment of urticaria in primary care. North Clin. Istanb..

[75] Schaefer P. (2017). Acute and Chronic Urticaria: Evaluation and Treatment. Am. Fam. Physician.

[76] Sabroe R.A., Greaves M.W. (1997). The pathogenesis of chronic idiopathic urticaria. Arch. Dermatol..

[77] Henry D., Ackerman M., Sancelme E., Finon A., Esteve E. (2020). Urticarial eruption in COVID-19 infection. J. Eur. Acad. Dermatol. Venereol..

[78] Recalcati S. (2020). Cutaneous manifestations in COVID-19: A first perspective. J. Eur. Acad. Dermatol. Venereol..

[79] Algaadi S.A. (2020). Urticaria and COVID-19: A review. Dermatol. Ther..

[80] De Giorgi V., Recalcati S., Jia Z., Chong W., Ding R., Deng Y., Scarfi F., Venturi F., Trane L., Gori A. (2020). Cutaneous manifestations related to coronavirus disease 2019 (COVID-19): A prospective study from China and Italy. J. Am. Acad. Dermatol..

[81] Dastoli S., Bennardo L., Patruno C., Nisticò S.P. (2020). Are erythema multiforme and urticaria related to a better outcome of COVID-19?. Dermatol. Ther..

[82] Jesenak M., Banovcin P., Diamant Z. (2020). COVID-19, chronic inflammatory respiratory diseases and eosinophils-Observations from reported clinical case series. Allergy.

[83] Rosenberg H.F., Foster P.S. (2021). Eosinophils and COVID-19: Diagnosis, prognosis, and vaccination strategies. Semin. Immunopathol..

[84] Ferastraoaru D., Hudes G., Jerschow E., Jariwala S., Karagic M., de Vos G., Rosenstreich D., Ramesh M. (2021). Eosinophilia in Asthma Patients Is Protective against Severe COVID-19 Illness. J. Allergy Clin. Immunol. Pract..

[85] Rodríguez-Jiménez P., Chicharro P., De Argila D., Muñoz-Hernández P., Llamas-Velasco M. (2020). Urticaria-like lesions in COVID-19 patients are not really urticaria—A case with clinicopathological correlation. J. Eur. Acad. Dermatol. Venereol..

[86] Zipursky J.S., Croitoru D. (2021). Urticaria and angioedema associated with SARS-CoV-2 infection. CMAJ.

[87] Hassan K. (2020). Urticaria and angioedema as a prodromal cutaneous manifestation of SARS-CoV-2 (COVID-19) infection. BMJ Case Rep..

[88] Najafzadeh M., Shahzad F., Ghaderi N., Ansari K., Jacob B., Wright A. (2020). Urticaria (angioedema) and COVID-19 infection. J. Eur. Acad. Dermatol. Venereol..

[89] Proietti I., Mambrin A., Bernardini N., Tolino E., Balduzzi V., Maddalena P., Marchesiello A., Michelini S., Volpe S., Skroza N. (2020). Urticaria in an infant with SARS-CoV-2 positivity. Dermatol. Ther..

[90] Morey-Olivé M., Espiau M., Mercadal-Hally M., Lera-Carballo E., García-Patos V. (2020). Cutaneous manifestations in the current pandemic of coronavirus infection disease (COVID 2019). An. Pediatr..

[91] Pagali S., Parikh R.S. (2021). Severe urticarial rash as the initial symptom of COVID-19 infection. BMJ Case Rep..

[92] Shanshal M. (2022). Low- dose systemic steroids, an emerging therapeutic option for COVID-19 related urticaria. J. Dermatolog. Treat..

[93] Sajjan V.V., Lunge S., Swamy M.B., Pandit A.M. (2015). Livedo reticularis: A review of the literature. Indian Dermatol. Online J..

[94] Verheyden M., Grosber M., Gutermuth J., Velkeniers B. (2020). Relapsing symmetric livedo reticularis in a patient with COVID-19 infection. J. Eur. Acad. Dermatol. Venereol..

[95] Khalil S., Hinds B.R., Manalo I.F., Vargas I.M., Mallela S., Jacobs R. (2020). Livedo reticularis as a presenting sign of severe acute respiratory syndrome coronavirus 2 infection. JAAD Case Rep..

[96] Agnihothri R., Fox L.P. (2021). Clinical Patterns and Morphology of COVID-19 Dermatology. Dermatol. Clin..

[97] Chand S., Rrapi R., Lo J.A., Song S., Gabel C.K., Desai N., Hoang M.P., Kroshinsky D. (2021). Purpuric ulcers associated with COVID-19: A case series. JAAD Case Rep..

[98] Pincelli M.S., Echavarria A., Criado P.R., Marques G.F., Morita T., Valente N., de Carvalho J.F. (2021). Livedo Racemosa: Clinical, Laboratory, and Histopathological Findings in 33 Patients. Int. J. Low Extrem. Wounds.

[99] Jamshidi P., Hajikhani B., Mirsaeidi M., Vahidnezhad H., Dadashi M., Nasiri M.J. (2021). Skin Manifestations in COVID-19 Patients: Are They Indicators for Disease Severity? A Systematic Review. Front. Med..

[100] Wysong A., Venkatesan P. (2011). An approach to the patient with retiform purpura. Dermatol. Ther..

[101] Daneshgaran G., Dubin D.P., Gould D.J. (2020). Cutaneous Manifestations of COVID-19: An Evidence-Based Review. Am. J. Clin. Dermatol..

[102] Marzano A.V., Genovese G., Fabbrocini G., Pigatto P., Monfrecola G., Piraccini B.M., Veraldi S., Rubegni P., Cusini M., Caputo V. (2020). Varicella-like exanthem as a specific COVID-19-associated skin manifestation: Multicenter case series of 22 patients. J. Am. Acad. Dermatol..

[103] Fernandez-Nieto D., Ortega-Quijano D., Jimenez-Cauhe J., Burgos-Blasco P., de Perosanz-Lobo D., Suarez-Valle A., Cortes-Cuevas J.L., Carretero I., Garcia-Del Real C., Fernandez-Guarino M. (2020). Clinical and histological characterization of vesicular COVID-19 rashes: A prospective study in a tertiary care hospital. Clin. Exp. Dermatol..

[104] Trellu L.T., Kaya G., Alberto C., Calame A., McKee T., Calmy A. (2020). Clinicopathologic Aspects of a Papulovesicular Eruption in a Patient With COVID-19. JAMA Dermatol..

[105] Mahé A., Birckel E., Merklen C., Lefèbvre P., Hannedouche C., Jost M., Droy-Dupré L. (2020). Histology of skin lesions establishes that the vesicular rash associated with COVID-19 is not ‘varicella-like’. J. Eur. Acad. Dermatol. Venereol..

[106] Villalon-Gomez J.M. (2018). Pityriasis Rosea: Diagnosis and Treatment. Am. Fam. Physician.

[107] Kutlu Ö., Metin A. (2020). Relative changes in the pattern of diseases presenting in dermatology outpatient clinic in the era of the COVID-19 pandemic. Dermatol. Ther..

[108] Merhy R., Sarkis A.S., Stephan F. (2021). Pityriasis rosea as a leading manifestation of COVID-19 infection. J. Eur. Acad. Dermatol. Venereol..

[109] Dursun R., Temiz S.A. (2020). The clinics of HHV-6 infection in COVID-19 pandemic: Pityriasis rosea and Kawasaki disease. Dermatol. Ther..

[110] Veraldi S., Spigariolo C.B. (2021). Pityriasis rosea and COVID-19. J. Med. Virol..

[111] Martín Enguix D., Salazar Nievas M.D.C., Martín Romero D.T. (2020). Pityriasis rosea Gibert type rash in an asymptomatic patient that tested positive for COVID-19. Med. Clin..

[112] Potekaev N.N., Zhukova O.V., Protsenko D.N., Demina O.M., Khlystova E.A., Bogin V. (2020). Clinical characteristics of dermatologic manifestations of COVID-19 infection: Case series of 15 patients, review of literature, and proposed etiological classification. Int. J. Dermatol..

[113] Sanchez A., Sohier P., Benghanem S., L’Honneur A.S., Rozenberg F., Dupin N., Garel B. (2020). Digitate Papulosquamous Eruption Associated with Severe Acute Respiratory Syndrome Coronavirus 2 Infection. JAMA Dermatol..

[114] Drago F., Ciccarese G., Rebora A., Parodi A. (2021). Human herpesvirus-6, -7, and Epstein-Barr virus reactivation in pityriasis rosea during COVID-19. J. Med. Virol..

[115] Welsh E., Cardenas-de la Garza J.A., Cuellar-Barboza A., Franco-Marquez R., Arvizu-Rivera R.I. (2021). SARS-CoV-2 spike protein positivity in pityriasis rosea-like and urticaria-like rashes of COVID-19. Br. J. Dermatol..

[116] Perna A., Passiatore M., Massaro A., Terrinoni A., Bianchi L., Cilli V., D’Orio M., Proietti L., Taccardo G., De Vitis R. (2021). Skin manifestations in COVID-19 patients, state of the art. A systematic review. Int. J. Dermatol..

[117] World Health Organization Multisystem Inflammatory Syndrome in Children and Adolescents with COVID-19. https://www.who.int/publications/i/item/multisystem-inflammatory-syndrome-in-children-and-adolescents-with-covid-19.

[118] Lu X., Zhang L., Du H., Zhang J., Li Y.Y., Qu J., Zhang W., Wang Y., Bao S., Li Y. (2020). SARS-CoV-2 Infection in Children. N. Engl. J. Med..

[119] Riphagen S., Gomez X., Gonzalez-Martinez C., Wilkinson N., Theocharis P. (2020). Hyperinflammatory shock in children during COVID-19 pandemic. Lancet.

[120] Centers for Disease Control and Prevention Information for Healthcare Providers about Multisystem Inflammatory Syndrome in Children (mis-C). https://www.cdc.gov/mis/misc/hcp/index.html?CDC_AA_refVal=https%3A%2F%2Fwww.cdc.gov%2Fmis%2Fhcp%2Findex.html.

[121] Dong Y., Mo X., Hu Y., Qi X., Jiang F., Jiang Z., Tong S. (2020). Epidemiology of COVID-19 among Children in China. Pediatrics.

[122] Licciardi F., Pruccoli G., Denina M., Parodi E., Taglietto M., Rosati S., Montin D. (2020). SARS-CoV-2-Induced Kawasaki-Like Hyperinflammatory Syndrome: A Novel COVID Phenotype in Children. Pediatrics.

[123] Godeau D., Petit A., Richard I., Roquelaure Y., Descatha A. (2020). An outbreak of severe Kawasaki-like disease at the Italian epicentre of the SARS-CoV-2 epidemic: An observational cohort study. Lancet.

[124] Hennon T.R., Penque M.D., Abdul-Aziz R., Alibrahim O.S., McGreevy M.B., Prout A.J., Schaefer B.A., Ambrusko S.J., Pastore J.V., Turkovich S.J. (2020). COVID-19 associated Multisystem Inflammatory Syndrome in Children (MIS-C) guidelines; a Western New York approach. Prog. Pediatr. Cardiol..

[125] Center for Disease Control and Prevention Multisystem Inflammatory Syndrome in Children (MIS-C) Associated with Coronavirus Disease 2019 (COVID-19). https://emergency.cdc.gov/han/2020/han00432.asp.

[126] Feldstein L.R., Rose E.B., Horwitz S.M., Collins J.P., Newhams M.M., Son M., Newburger J.W., Kleinman L.C., Heidemann S.M., Martin A.A. (2020). Multisystem Inflammatory Syndrome in U.S. Children and Adolescents. N. Engl. J. Med..

[127] Brumfiel C.M., DiLorenzo A.M., Petronic-Rosic V.M. (2021). Dermatologic manifestations of COVID-19-associated multisystem inflammatory syndrome in children. Clin. Dermatol..

[128] Shakeel S., Ahmad Hassali M.A. (2021). Post-COVID-19 Outbreak of Severe Kawasaki-like Multisystem Inflammatory Syndrome in Children. Malays. J. Med. Sci..

[129] Henderson L.A., Canna S.W., Friedman K.G., Gorelik M., Lapidus S.K., Bassiri H., Behrens E.M., Ferris A., Kernan K.F., Schulert G.S. (2020). American College of Rheumatology Clinical Guidance for Multisystem Inflammatory Syndrome in Children Associated with SARS-CoV-2 and Hyperinflammation in Pediatric COVID-19: Version 1. Arthritis Rheumatol..

[130] Wollina U., Karadağ A.S., Rowland-Payne C., Chiriac A., Lotti T. (2020). Cutaneous signs in COVID-19 patients: A review. Dermatol. Ther..

[131] Sachdeva M., Gianotti R., Shah M., Bradanini L., Tosi D., Veraldi S., Ziv M., Leshem E., Dodiuk-Gad R.P. (2020). Cutaneous manifestations of COVID-19: Report of three cases and a review of literature. J. Dermatol. Sci..

[132] Zaladonis A., Huang S., Hsu S. (2020). COVID toes or pernio?. Clin. Dermatol..

[133] Ladha M.A., Luca N., Constantinescu C., Naert K., Ramien M.L. (2020). Approach to Chilblains During the COVID-19 Pandemic. J. Cutan. Med. Surg..

[134] Rustin M.H., Newton J.A., Smith N.P., Dowd P.M. (1989). The treatment of chilblains with nifedipine: The results of a pilot study, a double-blind placebo-controlled randomized study and a long-term open trial. Br. J. Dermatol..

[135] Bosch-Amate X., Giavedoni P., Podlipnik S., Andreu-Febrer C., Sanz-Beltran J., Garcia-Herrera A., Alós L., Mascaró J.M. (2020). Retiform purpura as a dermatological sign of coronavirus disease 2019 (COVID-19) coagulopathy. J. Eur. Acad. Dermatol. Venereol..

[136] Brody G., Nguyen M.O., Foulad D.P., Rojek N.W. (2021). Retiform Purpura in the Setting of COVID-19: A Harbinger of Underlying Coagulopathy and Severe Disease Course. SKIN J. Cutan. Med..

[137] Shulman S.T. (2020). Pediatric Coronavirus Disease-2019-Associated Multisystem Inflammatory Syndrome. J. Pediatr. Infect. Dis. Soc..

[138] Cheung E.W., Zachariah P., Gorelik M., Boneparth A., Kernie S.G., Orange J.S., Milner J.D. (2020). Multisystem Inflammatory Syndrome Related to COVID-19 in Previously Healthy Children and Adolescents in New York City. JAMA.

[139] Son M., Murray N., Friedman K., Young C.C., Newhams M.M., Feldstein L.R., Loftis L.L., Tarquinio K.M., Singh A.R., Heidemann S.M. (2021). Multisystem Inflammatory Syndrome in Children—Initial Therapy and Outcomes. N. Engl. J. Med..

[140] Bursal Duramaz B., Yozgat C.Y., Yozgat Y., Turel O. (2020). Appearance of skin rash in pediatric patients with COVID-19: Three case presentations. Dermatol. Ther..

[141] Gianotti R., Recalcati S., Fantini F., Riva C., Milani M., Dainese E., Boggio F. (2020). Histopathological Study of a Broad Spectrum of Skin Dermatoses in Patients Affected or Highly Suspected of Infection by COVID-19 in the Northern Part of Italy: Analysis of the Many Faces of the Viral-Induced Skin Diseases in Previous and New Reported Cases. Am. J. Dermatopathol..

[142] Techasatian L., Lebsing S., Uppala R., Thaowandee W., Chaiyarit J., Supakunpinyo C., Panombualert S., Mairiang D., Saengnipanthkul S., Wichajarn K. (2020). The Effects of the Face Mask on the Skin Underneath: A Prospective Survey During the COVID-19 Pandemic. J. Prim. Care Community Health.

[143] Desai S.R., Kovarik C., Brod B., James W., Fitzgerald M.E., Preston A., Hruza G.J. (2020). COVID-19 and personal protective equipment: Treatment and prevention of skin conditions related to the occupational use of personal protective equipment. J. Am. Acad. Dermatol..

[144] Abdali S., Yu J. (2021). Occupational Dermatoses Related to Personal Protective Equipment Used During the COVID-19 Pandemic. Dermatol. Clin..

[145] Schwarz T., Kreiselmaier I., Bieber T., Thaci D., Simon J.C., Meurer M., Werfel T., Zuberbier T., Luger T.A., Wollenberg A. (2008). A randomized, double-blind, vehicle-controlled study of 1% pimecrolimus cream in adult patients with perioral dermatitis. J. Am. Acad. Dermatol..

[146] Wilkinson D.S., Kirton V., Wilkinson J.D. (1979). Perioral dermatitis: A 12-year review. Br. J. Dermatol..

[147] Two A.M., Wu W., Gallo R.L., Hata T.R. (2015). Rosacea: Part I. Introduction, categorization, histology, pathogenesis, and risk factors. J. Am. Acad. Dermatol..

[148] Park H., Del Rosso J.Q. (2011). Use of oral isotretinoin in the management of rosacea. J. Clin. Aesthet. Dermatol..

[149] Smart H., Opinion F.B., Darwich I., Elnawasany M.A., Kodange C. (2020). Preventing Facial Pressure Injury for Health Care Providers Adhering to COVID-19 Personal Protective Equipment Requirements. Adv. Skin Wound Care.

[150] Gefen A., Ousey K. (2020). Update to device-related pressure ulcers: SECURE prevention. COVID-19, face masks and skin damage. J. Wound Care.

[151] Dowdle T.S., Thompson M., Alkul M., Nguyen J.M., Sturgeon A. (2021). COVID-19 and dermatological personal protective equipment considerations. Bayl. Univ. Med Cent. Proc..

[152] Beiu C., Mihai M., Popa L., Cima L., Popescu M.N. (2020). Frequent Hand Washing for COVID-19 Prevention Can Cause Hand Dermatitis: Management Tips. Cureus.

[153] Cavanagh G., Wambier C.G. (2020). Rational hand hygiene during the coronavirus 2019 (COVID-19) pandemic. J. Am. Acad. Dermatol..

[154] Shanshal M., Ahmed H.S., Asfoor H., Salih R.I., Ali S.A., Aldabouni Y.K. (2021). Impact of COVID-19 on medical practice: A nationwide survey of dermatologists and health care providers in Iraq. Clin. Dermatol..

[155] Chiriac A.E., Wollina U., Azoicai D. (2020). Flare-up of Rosacea due to Face Mask in Healthcare Workers during COVID-19. Maedica.

[156] Yin Z.Q. (2020). Covid-19: Countermeasure for N95 mask-induced pressure sore. J. Eur. Acad. Dermatol. Venereol..

[157] Rundle C.W., Presley C.L., Militello M., Barber C., Powell D.L., Jacob S.E., Atwater A.R., Watsky K.L., Yu J., Dunnick C.A. (2020). Hand hygiene during COVID-19: Recommendations from the American Contact Dermatitis Society. J. Am. Acad. Dermatol..

[158] U.S. Food and Drug Administration COVID-19 Vaccines. https://www.fda.gov/emergency-preparedness-and-response/coronavirus-disease-2019-covid-19/covid-19-vaccines.

[159] GOV.UK Decision Conditions of Authorisation for COVID-19 Vaccine AstraZeneca (Regulation 174). https://www.gov.uk/government/publications/regulatory-approval-of-covid-19-vaccine-astrazeneca.

[160] Baraniuk C. (2021). COVID-19: What do we know about Sputnik V and other Russian vaccines?. BMJ.

[161] Montano D. (2022). Frequency and Associations of Adverse Reactions of COVID-19 Vaccines Reported to Pharmacovigilance Systems in the European Union and the United States. Front. Public Health.

[162] Centers for Disease Control and Prevention Allergic Reactions Including Anaphylaxis after Receipt of the First Dose of Moderna COVID-19 Vaccine—United States, December 21, 2020–January 10, 2021. https://www.cdc.gov/mmwr/volumes/70/wr/mm7004e1.htm.

[163] McLendon K., Sternard B.T. (2022). Anaphylaxis. StatPearls.

[164] Sobczak M., Pawliczak R. (2022). The risk of anaphylaxis behind authorized COVID-19 vaccines: A meta-analysis. Clin. Mol. Allergy.

[165] Iguchi T., Umeda H., Kojima M., Kanno Y., Tanaka Y., Kinoshita N., Sato D. (2021). Cumulative Adverse Event Reporting of Anaphylaxis After mRNA COVID-19 Vaccine (Pfizer-BioNTech) Injections in Japan: The First-Month Report. Drug Saf..

[166] Cabanillas B., Akdis C.A., Novak N. (2021). Allergic reactions to the first COVID-19 vaccine: A potential role of polyethylene glycol?. Allergy.

[167] Garvey L.H., Nasser S. (2021). Anaphylaxis to the first COVID-19 vaccine: Is polyethylene glycol (PEG) the culprit?. Br. J. Anaesth..

[168] Banerji A., Wickner P.G., Saff R., Stone C.A., Robinson L.B., Long A.A., Wolfson A.R., Williams P., Khan D.A., Phillips E. (2021). mRNA Vaccines to Prevent COVID-19 Disease and Reported Allergic Reactions: Current Evidence and Suggested Approach. J. Allergy Clin. Immunol. Pract..

[169] Wenande E.C., Skov P.S., Mosbech H., Poulsen L.K., Garvey L.H. (2013). Inhibition of polyethylene glycol-induced histamine release by monomeric ethylene and diethylene glycol: A case of probable polyethylene glycol allergy. J. Allergy Clin. Immunol..

[170] Bruusgaard-Mouritsen M.A., Johansen J.D., Garvey L.H. (2021). Clinical manifestations and impact on daily life of allergy to polyethylene glycol (PEG) in ten patients. Clin. Exp. Allergy.

[171] Sellaturay P., Nasser S., Ewan P. (2021). Polyethylene Glycol-Induced Systemic Allergic Reactions (Anaphylaxis). J. Allergy Clin. Immunol. Pract..

[172] Brandt N., Garvey L.H., Bindslev-Jensen U., Kjaer H.F., Bindslev-Jensen C., Mortz C.G. (2017). Three cases of anaphylaxis following injection of a depot corticosteroid with evidence of IgE sensitization to macrogols rather than the active steroid. Clin. Transl. Allergy.

[173] Zhou Z.H., Stone C.A., Jakubovic B., Phillips E.J., Sussman G., Park J., Hoang U., Kirshner S.L., Levin R., Kozlowski S. (2021). Anti-PEG IgE in anaphylaxis associated with polyethylene glycol. J. Allergy Clin. Immunol. Pract..

[174] Kozma G.T., Mészáros T., Vashegyi I., Fülöp T., Örfi E., Dézsi L., Rosivall L., Bavli Y., Urbanics R., Mollnes T.E. (2019). Pseudo-anaphylaxis to Polyethylene Glycol (PEG)-Coated Liposomes: Roles of Anti-PEG IgM and Complement Activation in a Porcine Model of Human Infusion Reactions. ACS Nano.

[175] Tihy M., Menzinger S., André R., Laffitte E., Toutous-Trellu L., Kaya G. (2021). Clinicopathological features of cutaneous reactions after mRNA-based COVID-19 vaccines. J. Eur. Acad. Dermatol. Venereol..

[176] Kempf W., Kettelhack N., Kind F., Courvoisier S., Galambos J., Pfaltz K. (2021). ‘COVID arm’—Histological features of a delayed-type hypersensitivity reaction to Moderna mRNA-1273 SARS-CoV2 vaccine. J. Eur. Acad. Dermatol. Venereol..

[177] Ramos C.L., Kelso J.M. (2021). “COVID Arm”: Very delayed large injection site reactions to mRNA COVID-19 vaccines. J. Allergy Clin. Immunol. Pract..

[178] Lindgren A.L., Austin A.H., Welsh K.M. (2021). COVID Arm: Delayed Hypersensitivity Reactions to SARS-CoV-2 Vaccines Misdiagnosed as Cellulitis. J Prim Care Community Health..

[179] Centers for Disease Control and Prevention COVID-19 Vaccine Distribution Allocations by Jurisdiction—Pfizer. https://data.cdc.gov/Vaccinations/COVID-19-Vaccine-Distribution-Allocations-by-Juris/saz5-9hgg.

[180] Wei N., Fishman M., Wattenberg D., Gordon M., Lebwohl M. (2021). “COVID arm”: A reaction to the Moderna vaccine. JAAD Case Rep..

[181] Kelso J.M., Coda A.B., Keating R.M., Vaccari D.M. (2021). “COVID Toes” After mRNA COVID-19 Vaccines. J. Allergy Clin. Immunol. Pract..

[182] Lesort C., Kanitakis J., Donzier L., Jullien D. (2021). Chilblain-like lesions after BNT162b2 mRNA COVID-19 vaccine: A case report suggesting that ‘COVID toes’ are due to the immune reaction to SARS-CoV-2. J. Eur. Acad. Dermatol. Venereol..

[183] Tan S.W., Tam Y.C., Oh C.C. (2021). Skin manifestations of COVID-19: A worldwide review. JAAD Int..

[184] Akdaş E., İlter N., Öğüt B., Erdem Ö. (2021). Pityriasis rosea following CoronaVac COVID-19 vaccination: A case report. J. Eur. Acad. Dermatol. Venereol..

[185] Burlando M., Herzum A., Cozzani E., Parodi A. (2021). Acute urticarial rash after COVID-19 vaccination containing Polysorbate 80. Clin. Exp. Vaccine Res..

[186] Hossain M.M., Tasnim S., Sultana A., Faizah F., Mazumder H., Zou L., McKyer E., Ahmed H.U., Ma P. (2020). Epidemiology of mental health problems in COVID-19: A review. F1000Research.

[187] Chaves C., Castellanos T., Abrams M., Vazquez C. (2018). The impact of economic recessions on depression and individual and social well-being: The case of Spain (2006–2013). Soc. Psychiatry Psychiatr. Epidemiol..

[188] Tapia Granados J.A., Christine P.J., Ionides E.L., Carnethon M.R., Diez Roux A.V., Kiefe C.I., Schreiner P.J. (2018). Cardiovascular Risk Factors, Depression, and Alcohol Consumption During Joblessness and During Recessions Among Young Adults in CARDIA. Am. J. Epidemiol..

[189] Beaglehole B., Mulder R.T., Frampton C.M., Boden J.M., Newton-Howes G., Bell C.J. (2018). Psychological distress and psychiatric disorder after natural disasters: Systematic review and meta-analysis. Br. J. Psychiatry..

[190] Reich A., Wójcik-Maciejewicz A., Slominski A.T. (2010). Stress and the skin. G. Ital. Dermatol. Venereol..

[191] Ferreira B.R., Jafferany M. (2021). Classification of psychodermatological disorders. J. Cosmet. Dermatol..

[192] Lv S., Wang L., Zou X., Wang Z., Qu B., Lin W., Yang D.A. (2021). Case of Acute Telogen Effluvium after SARS-CoV-2 Infection. Clin. Cosmet. Investig. Dermatol..

[193] Malkud S. (2015). Telogen Effluvium: A Review. J. Clin. Diagn. Res..

[194] Sharquie K.E., Jabbar R.I. (2021). COVID-19 infection is a major cause of acute telogen effluvium. Ir. J. Med. Sci..

[195] Mieczkowska K., Deutsch A., Borok J., Guzman A.K., Fruchter R., Patel P., Wind O., McLellan B.N., Mann R.E., Halverstam C.P. (2021). Telogen effluvium: A sequela of COVID-19. Int. J. Dermatol..

[196] Nair P.A., Badri T. (2022). Psoriasis.

[197] Cullen G., Kroshinsky D., Cheifetz A.S., Korzenik J.R. (2011). Psoriasis associated with anti-tumour necrosis factor therapy in inflammatory bowel disease: A new series and a review of 120 cases from the literature. Aliment. Pharmacol. Ther..

[198] Pugliese D., Guidi L., Ferraro P.M., Marzo M., Felice C., Celleno L., Landi R., Andrisani G., Pizzolante F., De Vitis I. (2015). Paradoxical psoriasis in a large cohort of patients with inflammatory bowel disease receiving treatment with anti-TNF alpha: 5-year follow-up study. Aliment. Pharmacol. Ther..

[199] Eickstaedt J.B., Killpack L., Tung J., Davis D., Hand J.L., Tollefson M.M. (2017). Psoriasis and Psoriasiform Eruptions in Pediatric Patients with Inflammatory Bowel Disease Treated with Anti-Tumor Necrosis Factor Alpha Agents. Pediatr. Dermatol..

[200] Ali E., Mohamed A., Abuodeh J., Albuni M.K., Al-Mannai N., Salameh S., Petkar M., Habas E. (2021). SARS-CoV-2 and guttate psoriasis: A case report and review of literature. Clin. Case Rep..

[201] Kuang Y., Shen M., Wang Q., Xiao Y., Lv C., Luo Y., Zhu W., Chen X. (2020). Association of outdoor activity restriction and income loss with patient-reported outcomes of psoriasis during the COVID-19 pandemic: A web-based survey. J. Am. Acad. Dermatol..

[202] Breuer K., Göldner F.M., Jäger B., Werfel T., Schmid-Ott G. (2015). Chronic stress experience and burnout syndrome have appreciable influence on health-related quality of life in patients with psoriasis. J. Eur. Acad. Dermatol. Venereol..

[203] Guertler A., Moellhoff N., Schenck T.L., Hagen C.S., Kendziora B., Giunta R.E., French L.E., Reinholz M. (2020). Onset of occupational hand eczema among healthcare workers during the SARS-CoV-2 pandemic: Comparing a single surgical site with a COVID-19 intensive care unit. Contact Dermat..

[204] Singh M., Pawar M., Bothra A., Choudhary N. (2020). Overzealous hand hygiene during the COVID 19 pandemic causing an increased incidence of hand eczema among general population. J. Am. Acad. Dermatol..

[205] Edison K.E., Ward D.S., Dyer J.A., Lane W., Chance L., Hicks L.L. (2008). Diagnosis, diagnostic confidence, and management concordance in live-interactive and store-and-forward teledermatology compared to in-person examination. Telemed. E-Health.

[206] Yeboah C.B., Harvey N., Krishnan R., Lipoff J.B. (2021). The Impact of COVID-19 on Teledermatology: A Review. Dermatol. Clin..

[207] Lee J.J., English J.C. (2018). Teledermatology: A Review and Update. Am. J. Clin. Dermatol..

[208] Wang R.H., Barbieri J.S., Nguyen H.P., Stavert R., Forman H.P., Bolognia J.L., Kovarik C.L., Group for Research of Policy Dynamics in Dermatology (2020). Clinical effectiveness and cost-effectiveness of teledermatology: Where are we now, and what are the barriers to adoption?. J. Am. Acad. Dermatol..

[209] Oakley A.M., Reeves F., Bennett J., Holmes S.H., Wickham H. (2006). Diagnostic value of written referral and/or images for skin lesions. J. Telemed. Telecare.

[210] Ríos-Yuil J.M. (2012). Correlación del Teleateneo con el Ateneo presencial de Dermatología en el diagnóstico de las patologías cutáneas. Actas Dermosifiliogr..

[211] Vidal-Alaball J., Garcia Domingo J.L., Garcia Cuyàs F., Mendioroz Peña J., Flores Mateo G., Deniel Rosanas J., Sauch Valmaña G. (2018). A cost savings analysis of asynchronous teledermatology compared to face-to-face dermatology in Catalonia. BMC Health Serv. Res..

[212] Snoswell C.L., Caffery L.J., Whitty J.A., Soyer H.P., Gordon L.G. (2018). Cost-effectiveness of Skin Cancer Referral and Consultation Using Teledermoscopy in Australia. JAMA Dermatol..

[213] Viola K.V., Tolpinrud W.L., Gross C.P., Kirsner R.S., Imaeda S., Federman D.G. (2011). Outcomes of referral to dermatology for suspicious lesions: Implications for teledermatology. Arch. Dermatol..

[214] Ferrándiz L., Ojeda-Vila T., Corrales A., Martín-Gutiérrez F.J., Ruíz-de-Casas A., Galdeano R., Álvarez-Torralba I., Sánchez-Ibáñez F., Domínguez-Toro J.M., Encina F. (2017). Internet-based skin cancer screening using clinical images alone or in conjunction with dermoscopic images: A randomized teledermoscopy trial. J. Am. Acad. Dermatol..

[215] Datta S.K., Warshaw E.M., Edison K.E., Kapur K., Thottapurathu L., Moritz T.E., Reda D.J., Whited J.D. (2015). Cost and Utility Analysis of a Store-and-Forward Teledermatology Referral System: A Randomized Clinical Trial. JAMA Dermatol..

[216] Mahendran R., Goodfield M.J., Sheehan-Dare R.A. (2005). An evaluation of the role of a store-and-forward teledermatology system in skin cancer diagnosis and management. Clin. Exp. Dermatol..

[217] Ferrandiz L., Moreno-Ramirez D., Nieto-Garcia A., Carrasco R., Moreno-Alvarez P., Galdeano R., Bidegain E., Rios-Martin J.J., Camacho F.M. (2007). Teledermatology-based presurgical management for nonmelanoma skin cancer: A pilot study. Dermatol. Surg..

[218] Conforti C., Giuffrida R., Dianzani C., Di Meo N., Zalaudek I. (2020). COVID-19 and psoriasis: Is it time to limit treatment with immunosuppressants? A call for action. Dermatol. Ther..

[219] Conforti C., Giuffrida R., Dianzani C., Di Meo N., Zalaudek I. (2020). Biologic therapy for psoriasis during the COVID-19 outbreak: The choice is to weigh risks and benefits. Dermatol. Ther..

[220] Galimberti F., McBride J., Cronin M., Li Y., Fox J., Abrouk M., Herbst A., Kirsner R.S. (2020). Evidence-based best practice advice for patients treated with systemic immunosuppressants in relation to COVID-19. Clin. Dermatol..

[221] Cheng S., Zhao Y., Wang F., Chen Y., Kaminga A.C., Xu H. (2021). Comorbidities’ potential impacts on severe and non-severe patients with COVID-19: A systematic review and meta-analysis. Medicine.

[222] Suchonwanit P., Leerunyakul K., Kositkuljorn C. (2020). Cutaneous manifestations in COVID-19: Lessons learned from current evidence. J. Am. Acad. Dermatol..

[223] Jimenez-Cauhe J., Ortega-Quijano D., de Perosanz-Lobo D., Burgos-Blasco P., Vañó-Galván S., Fernandez-Guarino M., Fernandez-Nieto D. (2020). Enanthem in Patients With COVID-19 and Skin Rash. JAMA Dermatol..

[224] Mercola J., Grant W.B., Wagner C.L. (2020). Evidence Regarding Vitamin D and Risk of COVID-19 and Its Severity. Nutrients.

[225] Entrenas Castillo M., Entrenas Costa L.M., Vaquero Barrios J.M., Alcalá Díaz J.F., López Miranda J., Bouillon R., Quesada Gomez J.M. (2020). Effect of calcifediol treatment and best available therapy versus best available therapy on intensive care unit admission and mortality among patients hospitalized for COVID-19: A pilot randomized clinical study. J. Steroid Biochem. Mol. Biol..

[226] Quesada-Gomez J.M., Entrenas-Castillo M., Bouillon R. (2020). Vitamin D receptor stimulation to reduce acute respiratory distress syndrome (ARDS) in patients with coronavirus SARS-CoV-2 infections: Revised Ms SBMB 2020_166. J. Steroid Biochem. Mol. Biol..

[227] Nogues X., Ovejero D., Pineda-Moncusí M., Bouillon R., Arenas D., Pascual J., Ribes A., Guerri-Fernandez R., Villar-Garcia J., Rial A. (2021). Calcifediol Treatment and COVID-19-Related Outcomes. J. Clin. Endocrinol. Metab..

[228] Uwitonze A.M., Razzaque M.S. (2018). Role of Magnesium in Vitamin D Activation and Function. J. Am. Osteopath. Assoc..

[229] Gallizzi R., Sutera D., Spagnolo A., Bagnato A.M., Cannavò S.P., Grasso L., Guarneri C., Nunnari G., Mazza F., Pajno G.B. (2020). Management of pernio-like cutaneous manifestations in children during the outbreak of COVID-19. Dermatol. Ther..

[230] Mahé A., Birckel E., Krieger S., Merklen C., Bottlaender L. (2020). A distinctive skin rash associated with coronavirus disease 2019?. J. Eur. Acad. Dermatol. Venereol..

[231] Iancu G.M., Solomon A., Birlutiu V. (2020). Viral exanthema as manifestation of SARS-CoV-2 infection: A case report. Medicine.

[232] Abuelgasim E., Dona A.C.M., Sondh R.S., Harky A. (2021). Management of urticaria in COVID-19 patients: A systematic review. Dermatol. Ther..

[233] van Damme C., Berlingin E., Saussez S., Accaputo O. (2020). Acute urticaria with pyrexia as the first manifestations of a COVID-19 infection. J. Eur. Acad. Dermatol. Venereol..

[234] McCullough P.A., Kelly R.J., Ruocco G., Lerma E., Tumlin J., Wheelan K.R., Katz N., Lepor N.E., Vijay K., Carter H. (2021). Pathophysiological Basis and Rationale for Early Outpatient Treatment of SARS-CoV-2 (COVID-19) Infection. Am. J. Med..

[235] McCullough P.A., Alexander P.E., Armstrong R., Arvinte C., Bain A.F., Bartlett R.P., Berkowitz R.L., Berry A.C., Borody T.J., Brewer J.H. (2020). Multifaceted highly targeted sequential multidrug treatment of early ambulatory high-risk SARS-CoV-2 infection (COVID-19). Rev. Cardiovasc. Med..

[236] Abou-Ismail M.Y., Diamond A., Kapoor S., Arafah Y., Nayak L. (2020). The hypercoagulable state in COVID-19: Incidence, pathophysiology, and management. Thromb Res..

[237] Magro C., Mulvey J.J., Berlin D., Nuovo G., Salvatore S., Harp J., Baxter-Stoltzfus A., Laurence J. (2020). Complement associated microvascular injury and thrombosis in the pathogenesis of severe COVID-19 infection: A report of five cases. Transl. Res..

[238] Zhou B., She J., Wang Y., Ma X. (2020). Venous thrombosis and arteriosclerosis obliterans of lower extremities in a very severe patient with 2019 novel coronavirus disease: A case report. J. Thromb. Thrombolysis.

[239] Manalo I.F., Smith M.K., Cheeley J., Jacobs R. (2020). A dermatologic manifestation of COVID-19: Transient livedo reticularis. J. Am. Acad. Dermatol..

[240] Risma K.A., Edwards K.M., Hummell D.S., Little F.F., Norton A.E., Stallings A., Wood R.A., Milner J.D. (2021). Potential mechanisms of anaphylaxis to COVID-19 mRNA vaccines. J. Allergy Clin. Immunol..

[241] Preissner K.T., Fischer S., Deindl E. (2020). Extracellular RNA as a Versatile DAMP and Alarm Signal That Influences Leukocyte Recruitment in Inflammation and Infection. Front. Cell Dev. Biol..

[242] Harris J.M., Chess R.B. (2003). Effect of pegylation on pharmaceuticals. Nat. Rev. Drug Discov..

